# ATTIC is an integrated approach for predicting A-to-I RNA editing sites in three species

**DOI:** 10.1093/bib/bbad170

**Published:** 2023-05-06

**Authors:** Ruyi Chen, Fuyi Li, Xudong Guo, Yue Bi, Chen Li, Shirui Pan, Lachlan J M Coin, Jiangning Song

**Affiliations:** College of Information Engineering, Northwest A&F University, Shaanxi 712100, China; The Peter Doherty Institute for Infection and Immunity, The University of Melbourne, VIC 3000, Australia; College of Information Engineering, Northwest A&F University, Shaanxi 712100, China; The Peter Doherty Institute for Infection and Immunity, The University of Melbourne, VIC 3000, Australia; College of Information Engineering, Northwest A&F University, Shaanxi 712100, China; Biomedicine Discovery Institute and Department of Biochemistry and Molecular Biology, Monash University, VIC 3800, Australia; Biomedicine Discovery Institute and Department of Biochemistry and Molecular Biology, Monash University, VIC 3800, Australia; School of Information and Communication Technology, Griffith University, QLD 4222, Australia; The Peter Doherty Institute for Infection and Immunity, The University of Melbourne, VIC 3000, Australia; Biomedicine Discovery Institute and Department of Biochemistry and Molecular Biology, Monash University, VIC 3800, Australia; Monash Data Futures Institute, Monash University, VIC 3800, Australia

**Keywords:** machine learning, ensemble learning, RNA modification, A-to-I editing, feature selection

## Abstract

A-to-I editing is the most prevalent RNA editing event, which refers to the change of adenosine (A) bases to inosine (I) bases in double-stranded RNAs. Several studies have revealed that A-to-I editing can regulate cellular processes and is associated with various human diseases. Therefore, accurate identification of A-to-I editing sites is crucial for understanding RNA-level (i.e. transcriptional) modifications and their potential roles in molecular functions. To date, various computational approaches for A-to-I editing site identification have been developed; however, their performance is still unsatisfactory and needs further improvement. In this study, we developed a novel stacked-ensemble learning model, ATTIC (A-To-I ediTing predICtor), to accurately identify A-to-I editing sites across three species, including *Homo sapiens*, *Mus musculus* and *Drosophila melanogaster*. We first comprehensively evaluated 37 RNA sequence-derived features combined with 14 popular machine learning algorithms. Then, we selected the optimal base models to build a series of stacked ensemble models. The final ATTIC framework was developed based on the optimal models improved by the feature selection strategy for specific species. Extensive cross-validation and independent tests illustrate that ATTIC outperforms state-of-the-art tools for predicting A-to-I editing sites. We also developed a web server for ATTIC, which is publicly available at http://web.unimelb-bioinfortools.cloud.edu.au/ATTIC/. We anticipate that ATTIC can be utilized as a useful tool to accelerate the identification of A-to-I RNA editing events and help characterize their roles in post-transcriptional regulation.

## INTRODUCTION

RNA editing is a post-transcriptional process that alters the genomic template by adding, deleting or replacing nucleotides, thereby increasing the complexity of RNAs to regulate gene expression [1]. In humans, the most prevalent RNA editing event is A-to-I RNA editing, i.e. the conversion of adenosine (A) to inosine (I) occurring in both coding and noncoding transcripts, which is medicated by ADARs (adenosine deaminases that act on RNAs) [2]. Throughout the entire catalytic process, ADARs bind double-stranded RNA structures and subsequently delaminate A to I, which is recognised as guanosine (G) by the cellular machinery [3, 4]. A-to-I RNA editing has been demonstrated to impact numerous critical genetic processes, including alternative splicing regulation, RNAi activity modification, RNA localisation, interference with microRNA function, circular RNA biogenesis and nuclear retention [3, 5–7]. Besides, A-to-I RNA editing has been linked to several human diseases, such as neurological, cancer, cardiovascular and carcinogenic diseases [5, 8–12]. Therefore, understanding A-to-I editing is critical for investigating molecular functions and developing targeted drugs. To date, tremendous effort has been made to identify A-to-I editing sites leveraging various techniques experimentally. High-throughput RNA sequencing is a powerful tool for detecting RNA editing events. It involves sequencing RNA samples and then comparing the resulting sequences to a reference genome. Any differences between the RNA samples and the reference genome can be identified and analysed to determine whether they represent RNA editing events. One major advantage of this approach is that it is relatively simple and straightforward. High-throughput RNA sequencing data can be easily obtained using standard laboratory techniques, and the resulting data can be analysed using a variety of software tools. Numerous A-to-I RNA editing sites in *Homo sapiens* and *Mus musculus* have been effectively characterised and curated in several public databases, such as DARNED, RADAR and REDIportal [4, 13–17]. However, there are also limitations to using high-throughput RNA sequencing for RNA editing detection. A major limitation is that this approach requires a high-quality reference genome—if a reference genome is not available or incomplete, it may be difficult or impossible to accurately identify RNA editing events. Additionally, high-throughput RNA sequencing data can be complex and difficult to analyse, particularly for large datasets [18–20].

**Table 1:** Summary of ML-based A-to-I RNA editing prediction tools

Tool^a^	Year	Algorithm	Evaluation strategy	Feature encoding scheme	Evaluation metrics	Software/web server availability^b^	Species
PAI [17]	2016	SVM	LOO, independent test	PseDNC, DPCP	S_n_, S_p_, ACC, MCC	Decommissioned	*D. melanogaster*
RDDpred [23]	2016	RF	Independent test	Sequencing features	NPV	Yes	*H. sapiens*, *M. musculus*, *D. melanogaster*
RED-ML [24]	2017	LR	5-fold CV	Sequencing features	AUROC, AUPRC, F_0.5_ score	Yes	*H. sapiens*
iRNA-AI [18]	2017	SVM	LOO, independent test	PseKNC, ND, NCP	S_n_, S_p_, ACC, MCC	Decommissioned	*H. sapiens*
iRNA-3typeA [19]	2018	SVM	LOO	PseKNC	S_n_, S_p_, ACC, MCC	Yes	*H. sapiens*, *M. musculus*
EPAI-NC [20]	2019	LD-SVM	LOO, independent test	Kmer, CKSNAP	S_n_, S_p_, ACC, MCC, AUROC, AUPRC, F_1_ score	Decommissioned	*D. melanogaster*
PRESa2i [21]	2020	DT	Independent test	Kmer, CKSNAP, statistical features	S_n_, S_p_, ACC, MCC, AUROC	Yes	*D. melanogaster*
iMRM [22]	2020	XGBoost	LOO	KNC, one-hot, ND, NCP, DPCP	S_n_, S_p_, ACC, MCC, AUROC	Yes	*H. sapiens*
TAE-ML [26]	2021	RF	5-fold CV	Sequencing features	S_n_, S_p_, ACC, Prec, F_1_ score	No	*H. sapiens*
RDDSVM [27]	2021	SVM	Independent test	Sequencing features	S_n_, S_p_, ACC	Yes	*H. sapiens*

*Notes:* SVM, support vector machine; RF, random forest; LR, logistic regression; LD-SVM, locally deep support vector machine; DT, decision tree; 5-fold CV, 5-fold cross-validation; LOO, leave-one-out cross-validation; PseDNC, pseudo nucleic acid composition; DPCP, dinucleotide physicochemical properties; PseKNC, pseudo *K*-tuple nucleotide composition; NCP, nucleotide chemical property; ND, nucleotide density; CKSNAP, composition of *k*-spaced nucleic acid pairs; Kmer, *k*mer composition; KNC, *k*-tuple nucleotide composition; NPV, negative predictive value; PPV, positive predictive value; ACC, accuracy; S_n_, sensitivity; S_p_, specificity; MCC, Matthew’s correlation coefficient; AUROC, the area under the receiver operating characteristic curve; AUPRC, the area under the precision-recall curve; Prec, precision

^a^URLs for the listed tools: PAI—http://lin.uestc.edu.cn/server/PAI; RDDpred—http://epigenomics.snu.ac.kr/RDDpred/; RED-ML—https://github.com/BGIRED/RED-ML; iRNA-AI—http://lin.uestc.edu.cn/server/iRNA-AI/; iRNA-3typeA—http://lin-group.cn/server/iRNA-3typeA/; EPAI-NC—http://epai-nc.info/; PRESa2i—http://brl.uiu.ac.bd/presa2i/index.php; iMRM—http://www.bioml.cn/XG_iRNA/home; https://github.com/liukeweiaway/iMRM; RDDSVM—https://github.com/huseyintac/RDDSVM.

^b^Yes—the publication is accompanied with a web server/tool, which is still functioning; decommissioned—the web server/tool is no longer available; no—the publication has no web server or tool.

*De novo* prediction tools offer an alternative approach to identifying RNA editing events. These tools are computational methods, such as machine learning (ML)-based algorithms to predict potential RNA editing sites based on sequence features and other factors. An advantage of *de novo* prediction tools is that they can be used even in the absence of a reference genome. In addition, these tools can be highly specific and can identify novel RNA editing events that may be missed by high-throughput RNA sequencing. However, *de novo* prediction tools also have some limitations. They typically rely on complex computational algorithms, and the accuracy of the predictions can be affected by a variety of factors, including the quality of the input data and the choice of parameters used by the algorithms. Therefore, it is desirable to develop appropriate algorithms to ensure the accuracy and reliability of these approaches.

To date, various ML-based predictors have been proposed for the computational identification of A-to-I RNA editing sites. These approaches have been summarised in Table 1, where we highlighted the corresponding ML algorithms, feature encoding schemes, performance evaluation metrics and strategy, web server/software availability and related species. Based on the support vector machine (SVM), Chen *et al.* proposed PAI to identify A-to-I RNA editing sites in *Drosophila melanogaster* by combining pseudo dinucleotide composition (PseDNC) with physicochemical characteristics in 2016 [21]. The following year, they shifted the research focus to *H. sapiens* and developed iRNA-AI by integrating chemical properties of nucleotides and nucleotide density (ND) to predict the A-to-I RNA editing [22]. Later, iRNA-3typeA was implemented to detect three types of RNA editing events in *H. sapiens* and *M. musculus*, including m^1^A, m^6^A and A-to-I RNA editing [23]. In 2019, Ahmad *et al*. designed EPAI-NC to discover A-to-I sites in *D. melanogaster* by employing both *k*mer compositions (Kmer) and *k*-spaced nucleic acid pair composition (CKSNAP) [24]. Subsequently, other algorithms started to emerge. Choyon *et al*. developed a decision tree (DT)-based model by combining Kmer, CKSNAP and other statistical features to predict A-to-I events in *D. melanogaster* [25]. Liu *et al*. created a novel computational predictor known as iMRM, which can simultaneously predict four separate RNA editing events in *H. sapiens*, *M. musculus* and *Saccharomyces cerevisiae*. However, iMRM only made identifying A-to-I RNA editing in *H. sapiens* possible. In contrast to previous work, iMRM was developed based on the eXtreme Gradient Boosting (XGBoost) algorithm combined with five different types of features and employed a feature selection method to enhance the stability and performance of the predictor [26]. In addition, RDDpred [27], RED-ML [28], DeepRed [29], TAE-ML [30], RDDSVM [31], and EditPredict [32] have been developed based on sequencing features and ML approaches.

**Figure 1:**
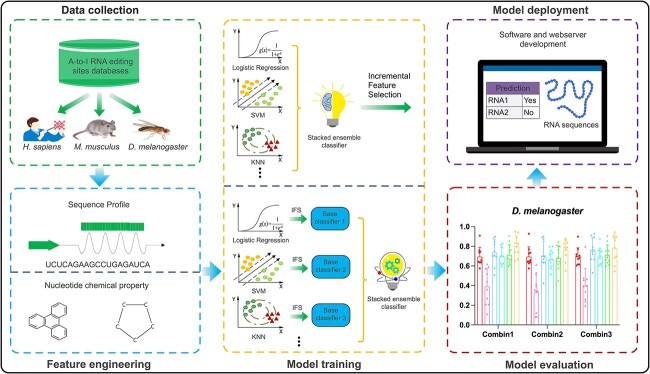
The overall framework of ATTIC.

Despite the computational advances in A-to-I editing prediction, these approaches have several limitations. First, an accurate, multi-species platform for predicting A-to-I RNA editing sites is lacking. Most of the existing methods can only predict A-to-I sites in *D. melanogaster* (e.g. PAI, EPAI-NC and PRESa2i) and cannot perform accurate predictions for other species. A-to-I editing is a complex biological process that can be influenced by various factors, including species-specific factors such as the expression level of ADAR enzymes and their isoforms and RNA editing site accessibility and regulation. The A-to-I editing level and patterns can vary across different species [4, 33–35]. Second, most existing tools rely on a single classifier or a few ML algorithms, which limits the prediction performance. For instance, PAI, iRNA-Ai and iRNA-3typeA all employed SVM to construct their models and only achieved relatively poor performance. Last but not least, the existing methods lacked a comprehensive profiling of A-to-I RNA modification sites and an adequate interpretation of their prediction results, such as iMRM and iRNA-3typeA.

To address these limitations, herein we proposed a novel stacking ensemble learning predictor, ATTIC (A-To-I ediTing predICtor), to accurately identify A-to-I RNA editing sites from RNA sequences in multiple species, including *H. sapiens*, *M. musculus* and *D. melanogaster*, based on our elaborate analysis of 37 different types of RNA sequence features and evaluation of 14 widely applied ML algorithms. We then constructed a series of stacked ensemble learning models based on selecting the most informative RNA sequence features and the most effective ML algorithms for specific species. Leave-one-out cross-validation (LOOCV) test results demonstrated that ATTIC significantly improves the prediction performance across all three species compared with state-of-the-art predictors. Furthermore, the Shapley Additive exPlanation algorithm (SHAP) was employed to interpret and highlight the most important features used in ATTIC, improving the model interpretability of ATTIC.

## MATERIALS AND METHODS

### The overall framework of ATTIC

Figure 1 shows the workflow of designing and evaluating ATTIC, including five major steps: data collection, feature engineering, model training, model evaluation and model deployment. In the first step, we retrieved and collected the A-to-I editing sites for three species, including *H. sapiens*, *M. musculus* and *D. melanogaster*. In the second step, we extracted 37 different types of RNA sequence features and employed 14 widely used ML algorithms to select the optimal feature encoding schemes for each species. In the third step, we built a set of ensemble models by combining various base classifiers with the stacking method and optimized the model with different feature selection strategies. In the fourth step, we comprehensively assessed the improved stacked models via LOOCV and independent tests and benchmarked ATTIC against existing state-of-the-art A-to-I editing site predictors. Finally, a web server and a stand-alone tool of ATTIC were implemented in order to provide convenient and high-throughput predictions of A-to-I editing sites.

### Data collection

In this study, we integrated the datasets from multiple previous studies. Specifically, PAI [21], EPAI-NC [24], PRESa2i [25], PAI-SAE [36] and iAI-DSAE [37] were all trained and assessed using the *D. melanogaster* dataset from St Laurent *et al*.’s work [38]. All these predictors detected A-to-I RNA editing by incorporating computational prediction and experimental validation. In addition, several tools have been developed to predict A-to-I RNA editing sites for *H. sapiens*, such as iRNA-Ai [22], iMRM [26] and MultiRM [39], whose datasets were derived from DARNED [13]. Likewise, originating from DARNED, iRNA-3typeA is the only tool available for identifying A-to-I RNA editing sites in *M. musculus* [23]. Relying on the aforementioned database and sequencing data, Chen *et al.* [21–23] constructed all three datasets successively by applying the CD-HIT program [40] to remove redundant sequences with the 60% sequence similarity threshold. Therefore, we constructed our benchmark datasets for *H. sapiens* (denoted as H), *M. musculus* (denoted as M) and *D. melanogaster* (denoted as D) from these previous studies. More specifically, H was collected from ‘iMRM’, M from ‘iRNA-3typeA’ and D from ‘PAI’ [21, 23, 26], respectively. As shown in Table 2, H_3000, M_831 and D_125 are three benchmark datasets with 3000, 831 and 125 positive samples, respectively. Since small datasets might experience overfitting issues during training, we also further utilized an independent test dataset D_300 to prove the model’s prediction ability for *D. melanogaster.* Based on Yu and colleagues’ work, Chen *et al*. constructed D_300 which contains 300 positive samples [21, 41]. All positive sequence samples are 51 nt sequences with an A at the centre in datasets H_3000, D_125 and D_300, while the positive samples are 41 nt sequences with an A in the centre in dataset M_831. It should be noted that all these datasets contain the same number of negative samples as the positives. The datasets used in this study are provided in Supplementary Data available online at http://bib.oxfordjournals.org/.

**Table 2:** Detailed information for the benchmark datasets used in ATTIC

Species	Datasets	Sequence length	The number of positive samples	The number of negative samples	The total number of samples	Source
*H. sapiens*	H_3000	51	3000	3000	6000	[22]
*M. musculus*	M_831	41	831	831	1662	[19]
*D. melanogaster*	D_125	51	125	119	244	[17]
	D_300	51	300	–	300	[17]

### Feature engineering

The feature representations have a significant impact on the performance of ML models. In order to discover the effective feature encoding schemes, we extracted 37 RNA sequence encoding methods using our iFeatureOmega package [42]. RNA sequences are composed of four types of nucleic acids (A, C, U and G). Therefore, there are 16 dinucleotide combinations theoretically denoted as AA, AC, AG, AU, CA, …, and UU. After the rough assessment of the 37 encoding methods using 10-fold CV, we selected six to eight encodings according to our preliminary results for each species based on different feature groups, including nucleic acid composition group, pseudo nucleic acid composition group, residue composition group and physicochemical property group. Through feature engineering, RNA sequences were converted to a fixed-length numerical feature vector. For *H. sapiens*, we used features of the enhanced nucleic acid composition (ENAC) and NCP from the nucleic acid composition group, position specific of two nucleotides (PS2), and binary from the residue composition group. For *M. musculus*, we also applied features of NCP and ENAC from the nucleic acid composition group, binary and dinucleotide binary encoding (DBE) from the residue composition group as well as DPCP type2 (DPCP2) from the physicochemical property group. Moreover, five features were utilized for *D. melanogaster*, including CKSNAP, Kmer and adaptive skip dinucleotide composition (ASDC) from the nucleic acid composition group, PseDNC from the pseudo nucleic acid composition and DPCP from the physicochemical property group. All feature encoding methods used in this study from different groups are given in the following section, and encodings used for comparison refer to Supplementary Data available online at http://bib.oxfordjournals.org/.

#### Group 1: nucleic acid composition features

##### *k*mer composition

Kmer denotes the frequency of occurrence of *k* neighbouring nucleic acids in an RNA sequence [43, 44]. *K* can be denoted with different *k* values (*k* = 1, 2, 3, 4 …). In particular, *k* was set to 2 (di-), 3 (tri-), 4 (tetra-) and 5 (penta-nucleotide composition) in this study. Taking *k* = 4 as an example, *k*mer can be calculated as follows:


(1) \begin{equation*} f(t)=\frac{N(t)}{N},t\in \left\{\text{AAAA},\text{AAA}\right.\left.\text{C},\text{AAA}\text{G},..,\text{UUUU}\right\}, \end{equation*}

where $N(t)$ is the number of *k*mer type $t$, and $N$ is the length of the nucleotide sequence.

##### Composition of *k*-spaced nucleic acid pairs

When using *k* nucleic acids (*k* = 0, 1, …, 5) separate nucleic acid pairs, CKSNAP refers to the frequency of those separated nucleic acid pairs [45]. Taking *k* = 2 as an example, there are 16 pairs of nucleic acids that are separated by zero, one and two separately. Then, the feature vector of CKSNAP can be calculated as follows:


(2) \begin{equation*} {\left(\frac{m_{\text{AA}}}{m_{\text{Total}}},\frac{m_{\text{AC}}}{m_{\text{Total}}},\frac{m_{\text{AG}}}{m_{\text{Total}}},\dots, \frac{m_{\text{UU}}}{m_{\text{Total}}}\right)}_{16}. \end{equation*}

In formula (2), the value of each descriptor indicates the composition of the nucleic acid pair matching it in the nucleotide sequence. For example, if the nucleic acid pair AA occurs $m$ times in the nucleotide sequence, its composition is ${m}_{\text{AA}}$ divided by the total number of 2-spaced nucleic acid pairs in the nucleotide sequence (${m}_{\text{Total}}$). For different *k*, the values of the total number are *P* − *k* − 1, where *P* is the length of a nucleotide sequence. In this study, *k* was set to 2, and we finally obtained a 48 (16$\times$3)-dimensional feature vector.

##### Adaptive skip dinucleotide composition

ASDC considers correlation information between neighbouring nucleotides and between intervening nucleotides [46]. For a given RNA sequence, the feature vector of ASDC is explained as follows:


(3) \begin{equation*} \text{ASDC}=\left({f}_{\nu 1},{f}_{\nu 2},{f}_{\nu 3},\dots \dots, {f}_{\nu 16}\right). \end{equation*}

In formula (3), *f_vi_* is defined as:


(4) \begin{equation*} {f}_{\nu i}=\frac{\sum_{g=1}^{L-1}{O}_i^g}{\sum_{i=1}^{16}\sum_{g=1}^{L-1}{O}_i^g}, \end{equation*}

where *L* denotes the sequence length and ${O}_i^g$ represents the occurrence number of the *i*-th (*i* = 1, 2, …, 16) *g*-gap (*g* = 1, 2, …, *L* − 1) dinucleotide. Hence, ${f}_{\nu i}$ denotes the occurrence frequency of all possible dinucleotides (i.e. AA, AC, AG, …, UU).

##### Enhanced nucleic acid composition

ENAC calculates the NAC based on a fixed-length sequence window that slides continuously from the 5′ to 3′ terminal of the nucleotide sequence and can be used to encode nucleotide sequences of equal length. Therefore, the composition of the enhanced nucleic acid can be calculated as


(5) \begin{align*} Q=\left[\frac{N_{A,{\text{win}}_1}}{k},\frac{N_{G,{\text{win}}_1}}{k},\frac{N_{C,{\text{win}}_1}}{k},\frac{N_{U,{\text{win}}_1}}{k},\frac{N_{A,{\text{win}}_2}}{k},\right.\nonumber\\\left.\frac{N_{G,{\text{win}}_2}}{k},\dots, \frac{N_{G,{\text{win}}_{L-k+1}}}{k},\frac{N_{G,{\text{win}}_{L-k+1}}}{k}\right]. \end{align*}

In this formula, *k* signifies the sliding window’s size, ${N}_{t,p}$denotes the number of nucleotides A in the sliding window *p*, *t*  $\epsilon$[A, C, G, U], and *p* = 1, 2, …, *L* − *k* + 1 denotes the sliding window’s position. For the proposed ATTIC method, the sliding window size *k* was set to 2, with a 200 (= 4 × 50)-dimensional feature vector.

##### Nucleotide chemical property

Each nucleotide in the RNA sequence has a unique chemical structure and chemical structure-binding properties [47]. These chemical characteristics of different types of nucleotides can be categorized into three different groups (Table 3).

**Table 3:** The chemical properties of different types of nucleotides in RNA sequences

Chemical property	Class	Nucleotides
Ring structure	Purine	A, G
	Pyrimidine	C, U
Functional group	Amino	A, C
	Keto	G, U
Hydrogen bond	Strong	C, G
	Weak	A, U

To encode these chemical qualities in RNA, three coordinates (*x*, *y*, *z*) were created to represent three chemical groups and give them values of 1 or 0. Thus, the sequence’s nucleotide *s_i_* = (*x_i_*, *y_i_*, *z_i_*) may be represented as follows:


(6) \begin{equation*} {x}_i=\left\{\begin{array}{@{}c}1\ \text{if}\ {s}_i\in \left\{\text{A},\text{G}\right\}\\{}\ 0\ \text{if}\ {s}_i\in \left\{\text{C},\text{U}\right\}\end{array}\right.\kern-.5pc,\\{y}_i=\left\{\begin{array}{@{}c}1\ \text{if}\ {s}_i\in \left\{\text{A},\text{C}\right\}\\{}\ 0\ \text{if}\ {s}_i\in \left\{\text{G},\text{U}\right\}\end{array}\right.\kern-.5pc,\\{z}_i=\left\{\begin{array}{@{}c}1\ \text{if}\ {s}_i\in \left\{\text{A},\text{U}\right\}\\{}\ 0\ \text{if}\ {s}_i\in \left\{\text{C},\text{G}\right\}\end{array}\right.\kern-.5pc,\end{equation*}

where the coordinate value of each nucleotide is determined by the nucleotide’s chemical properties: A was represented by coordinates (1, 1, 1), C was represented by coordinates (0, 1, 0), G was represented by coordinates (1, 0, 0) and U was represented by coordinates (0, 0, 1).

#### Group 2: physicochemical property features

##### Dinucleotide physicochemical properties

There are a total of 16 dinucleotide combinations theoretically. Each of these has different physicochemical properties for the formulation of pseudo dinucleotide composition, which are defined as


(7) \begin{equation*} \text{DPCP}(i)=f(i)\times \text{dpcp}(i), \end{equation*}

where *f* is the frequency of the dinucleotide *i*, and dpcp(*i*) is one of the *i*-th dinucleotide’s physicochemical qualities. In this work, DPCP was represented as a 96-dimensional vector (16 dinucleotides$\times$6 physicochemical characteristics), and the specific physicochemical properties include ‘rise’, ‘roll’, ‘shift’, ‘slide’, ‘tilt’ and ‘twist’.

##### DPCP type2

Different from DPCP, DPCP2 can be encoded as a matrix as follows:


(8) \begin{equation*} V=\left[\begin{array}{ccc}{\text{PC}}^1\left({N}_1{N}_2\right)& \cdots & {\text{PC}}^1\left({N}_{L-1}{N}_L\right)\\{}\vdots & \dots & \vdots \\{}{\text{PC}}^j\left({N}_1{N}_2\right)& \cdots & {\text{PC}}^j\left({N}_{L-1}{N}_L\right)\end{array}\right], \end{equation*}

where ${\text{PC}}^j\left({N}_{L-1}{N}_L\right)$represents the *j*-th physicochemical dinucleotide value of the dinucleotide ${N}_{L-1}{N}_L$, and *L* is the RNA sequence’s length [42]. ATTIC utilized the physicochemical properties of the ‘rise’, ‘roll’, ‘shift’ and ‘slide’.

#### Group 3: pseudo nucleic acid composition

##### Pseudo nucleic acid composition

In light of the successful use of pseudo amino acid composition (PseAAC) for predicting protein and peptide sequences, the researchers expanded its key concept to pseudo nucleic acid composition (PseNAC) and successfully applied it to the prediction of RNA and DNA sequences [48]. Chen *et al*. [49] proposed the PseDNC coding scheme, which is based on the PseAAC algorithm and incorporates contiguous local and global sequence-order information into the nucleotide sequence’s feature vector [37]. The PseDNC encoding is defined:


(9) \begin{equation*} \text{PseDNC}={\left[{d}_1,{d}_2,\dots, {d}_{16},{d}_{16+1},{d}_{16+2},\dots, {d}_{16+\lambda}\right]}^T, \end{equation*}

where ${d}_k$ is defined as


(10) \begin{equation*} {d}_k=\left\{\begin{array}{@{}c}\frac{f_k}{\sum_{i=1}^{16}{f}_i+w\sum_{j=1}^{\lambda }{\theta}_j}\left(1\le k\le 16\right)\\ {}\frac{w{\theta}_{k-16}}{\sum_{i=1}^{16}{f}_i+w\sum_{j=1}^{\lambda }{\theta}_j}\left(17\le k\le 16+\lambda \right)\end{array}\right.. \end{equation*}

In formula (10), *f_k_* (*k* = 1, 2, …, 16) is the normalized occurrence frequency of dinucleotide in the nucleotide sequence, $\lambda$ represents the highest counted rank (or tie) of the correlation along the nucleotide sequence, *w* is a weight factor ranging from 0 to 1, and *θ_j_* (*j* = 1, 2, …, *λ*) is the *j*-tier correlation factor and is defined as follows:


(11) \begin{equation*} \left\{\begin{array}{@{}c}{\theta}_1=\frac{1}{L-2}\ \sum_{i=1}^{L-2}\theta \left({R}_i{R}_{i+1,}{R}_{i+1}{R}_{i+2}\right)\\ {}{\theta}_2=\frac{1}{L-3}\ \sum_{i=1}^{L-3}\theta \left({R}_i{R}_{i+1,}{R}_{i+2}{R}_{i+3}\right)\\ {}{\theta}_3=\frac{1}{L-4}\ \sum_{i=1}^{L-4}\theta \left({R}_i{R}_{i+1,}{R}_{i+3}{R}_{i+4}\right)\ \left(\lambda <L\right)\\{}\cdots \\{}{\theta}_{\lambda }=\frac{1}{L-1-\lambda }\ \sum_{i=1}^{L-1-\lambda}\theta \left({R}_i{R}_{i+1,}{R}_{i+\lambda }{R}_{i+\lambda +1}\right)\end{array}\right.\kern-6pt, \end{equation*}

where the correlation function is defined:


(12) \begin{equation*} \theta \left({R}_i{R}_{i+1,}{R}_{j+1}{R}_{j+1}\right)=\frac{1}{\mu}\sum_{u=1}^{\mu }{\left[{P}_u\left({R}_i{R}_{i+1}\right)-{P}_u\left({R}_j{R}_{j+1}\right)\right]}^2. \end{equation*}

In formula (12), $\mu$ is the number of physicochemical indices. Six indices (‘rise’, ‘roll’, ‘shift’, ‘slide’, ‘tilt’ and ‘twist’) were set as the default indices for RNA. ${P}_u\left({R}_i{R}_{i+1}\right)$ denotes the number of the *u*-th (*u* = 1, 2, …, *μ*) physicochemical index of the dinucleotide ${R}_i{R}_{i+1}$ at position *i*, while ${P}_u\left({R}_j{R}_{j+1}\right)$represents the equivalent value of the dinucleotide ${R}_j{R}_{j+1}$ at position *j*. In this study, the value of *λ* was set to 2.

#### Group 4: residue composition

Binary, DBE and PS2 are three widely used residue composition-based encodings in bioinformatic studies [50–53]. Binary encoding refers to transforming a single category variable into several variables, each of which can take on the value of 0 or 1. It encodes a nucleotide acid as a 4-dimensional binary vector, where A is encoded by (1, 0, 0, 0), C is encoded by (0, 1, 0, 0), G is encoded by (0, 0, 1, 0) and U was encoded by (0, 0, 0, 1), respectively. Therefore, a 41 nt RNA sequence can be transformed into a 164-dimensional feature vector (41 × 4 dimensions) [45]. DBE incorporates information about the location of the dinucleotide in the sequence [50]. Each dinucleotide is represented using a 4-dimensional 0/1 vector. For instance, AA is encoded as (0, 0, 0, 0), AT as (0, 0, 0, 1), AC as (0, 0, 1, 0) and GG as (1, 1, 1, 1). In PS2, as there are 16 (4$\times$4) pairs of adjacent pairwise nucleotides (e.g. AA, AC, AG, AU), a single variable representing one of them is encoded into 16 binary variables. For example, AA is encoded by (1000000000000000) and AAC is encoded by (10000000000000000100000000000000) [51, 54].

### Stacking ensemble learning

Stacking ensemble learning methods have been widely used to improve the generalization performance of single algorithms in bioinformatics tasks [55–59]. The specific step of the stacking strategy is to select a collection of base classifiers and then use their outputs as the input for a meta-classifier model. In this study, A-to-I RNA editing prediction is a binary classification task, i.e. the model was designed to predict whether a given RNA sequence contains A-to-I RNA editing sites. To determine the base classifiers for each species, we first comprehensively measured the prediction performance of 14 popular ML algorithms for each of the RNA encoding schemes aforementioned, including Catboost [60], Random Forest (RF), Extra Trees (ET) [61], Gradient Boosting Decision Tree [62], SVM, Ridge, Linear Discriminant Analysis (LDA), Naïve Bayes (NB), Logistic Regression (LR), Adaptive Boosting (AdaBoost), DT, *K*-nearest neighbours (KNN), Quadratic Discriminant Analysis and Light Gradient Boosting Machine (LightGBM) [63]. By building these classifiers based on the Scikit-learn package in Python [64] with ten times 10-fold CV, we then selected six base classifiers that have good prediction performance in all three species. A brief introduction of these six base classifiers is presented in Supplementary Data available online at http://bib.oxfordjournals.org/.

### Feature selection

The high-dimensional feature sets probably contain irrelevant or redundant information, which could influence the performance and efficiency of the model training [65]. Therefore, it is essential to identify informative features to enhance the model performance [66, 67]. In this study, we employed two amended incremental feature selection (IFS) strategies to improve the predictive performance of trained models and selected the optimal design for each species-specific model. The two critical factors for IFS strategies are the feature ranking method and the base classifier, as IFS ranks and selects the features according to the classification performance [68]. The detailed procedures of these two IFS strategies are provided in Algorithms 1 and 2, respectively. For each species, we randomly divided the benchmark dataset into two subsets, one for training and the other for performance evaluation. In Algorithm 1, ${C}_n$ is the classifier set, and ${F}_m$ is the original feature set. For each classifier ${c}_i$ in the ${C}_n$, the features in the ${F}_m$ were sorted in descending order according to the feature importance, and the sorted feature set was denoted as ${F}_m^{\prime }=\left({f}_1^{\prime },{f}_2^{\prime },\dots, {f}_m^{\prime}\right)$ (step 2). Then, we selected the top *k* features from ${F}_m^{\prime }$ to conduct 10 times 10-fold CV and recorded the average MCC and area under the receiver operating characteristic curve (AUC) values (steps 3–6). Subsequently, we determined the optimal feature subset for ${c}_i$ by the maximum MCC and AUC value (step 7). If more than one subset achieved the best MCC, we determined the optimal one by comparing their AUC values. We then retrained the base classifier ${c}_i^{\prime }$ by using the optimal feature ${F}_i^{\prime }$ at step 8. ${C}_n^{\prime }$ at step 10 is the base classifier set optimized after IFS, and $F$ is the union optimal feature set. Finally, we ensembled the base classifiers ${c}_i^{\prime }$ to build the stacked model $stacked1$ and return it at steps 12–13. As for Algorithm 2, the original feature set ${F}_m^{\prime }$ is ranked based on the feature importance generated by the best classifier in ${F}_m^{\prime }$. Similarly, in steps 2–6, we selected the top *k* features of ${F}_m^{\prime }$as ${f}_k^{\prime }$ and trained a stacked ensemble model $stackedModel$ based on ${C}_n$. Then, we evaluated the performance of $stackedModel$ by 10 times 10-fold CV and record the average MCC and AUC values. Subsequently, we used the $optimalFeatureSet\left(\right)$ function to get the optimal feature subset $F$ and train the stacked model $stacked2$ at steps 7–9. We used these two algorithms to train two different stacked ensemble models for each species, and the model with better predictive performance was used as the optimal model.

**Algorithm 1:** Incremental feature selection strategy 1

** Input**: classifier set ${C}_n=\left({c}_1,{c}_2,\dots, {c}_n\right)$; original feature set ${F}_m=\left({f}_{1,}{f}_2,\dots, {f}_m\right)$ ** Output**: the optimal stacked ensemble model $stacked1$ 1: **for** classifiers ${c}_i$ in ${C}_n$ **do:** 2: rank the features in descending order $\to$ ${F}_m^{\prime }=\left({f}_1^{\prime },{f}_2^{\prime },\dots, {f}_m^{\prime}\right)$
3: **for** $k\ \epsilon\ \left(1,m\right)$ **do:** 4: ${F}_k^{\prime }={F}_m^{\prime}\left[1:k\right]$ 5: ${MCCs}_i\left[\right],{AUCs}_i\left[\right]= CV\_ test\left({c}_i,{f}_k^{\prime }, fold=10, times=10\right)$ 6: **End for** 7: ${F}_i^{\prime }=\max \left({MCCs}_i,{AUCs}_i\right)$ 8: ${c}_i^{\prime }= classifier. fit\left({c}_i,{F}_i^{\prime}\right)$ 9: **End for** 10: ${C}_n^{\prime }=\left({c}_1^{\prime },{c}_2^{\prime },\dots, {c}_n^{\prime}\right)$ 11: $F=\bigcup_{i=1}^n{F}_i^{\prime }$ 12: $stacked1= stacking. fit\left({C}_n^{\prime },F\right)$ 13: **return** $stacked1$

**Algorithm 2:** Incremental feature selection strategy 2

** Input**: the classifier set ${C}_n=\left({c}_1,{c}_2,\dots, {c}_n\right)$; original feature set ${F}_m$ = $\left({f}_{1,}{f}_2,\dots, {f}_m\right)$ ** Output**: the optimal stacked ensemble model $stacked2$ 1: rank the features in descending order using *optimalClassifier. feature_importance* $\to{F}_m^{\prime }=\left({f}_1^{\prime },{f}_2^{\prime },\dots, {f}_m^{\prime}\right)$
2: **for** $k\epsilon \left(1,m\right)$ **do:** 3: ${f}_k^{\prime }={F}_m^{\prime}\left[1:k\right]$ 4: $stackedModel= stacking. fit\left({C}_n,{f}_k^{\prime}\right)$ 5: $MCCs\left[\right], AUCs\left[\right]= CV\_ test\left( stackedModel,{f}_k^{\prime }, fold=10, times=10\right)$ 6: **End for** 7: $F= optimalFeatureSet\left( MCCs, AUCs\right)$ 8: $stacked2= stacking. fit\left({C}_n,F\right)$ 9: **return** $stacked2$

### Performance evaluation

In this study, we evaluated the model performance of ATTIC using three cross-validation strategies, including (i) the 10-fold cross-validation (CV) test, (ii) the leave-one-out (LOO) test and (iii) the independent test. The 10-fold CV test was used to select the optimal features and base classifiers. Then, we conducted LOO and independent tests to evaluate and benchmark the predictive performance of ATTIC with existing methods. As for performance evaluation metrics, we employed Recall, Specificity (Sp), Matthew’s correlation coefficient (MCC), Accuracy (ACC), Precision and F_1_ score. These performance metrics are defined as:


(13) \begin{equation*} \text{Recall}=\frac{\text{TP}}{\text{TP}+\text{FN}} \end{equation*}

(14) \begin{equation*} \text{Sp}=\frac{\text{TN}}{\text{TN}+\text{FP}} \end{equation*}

(15) \begin{equation*} \text{Acc}=\frac{\text{TP}+\text{TN}}{\text{TP}+\text{TN}+\text{FP}+\text{FN}} \end{equation*}

(16) \begin{equation*} \text{MCC}=\frac{\left(\text{TP}\times \text{TN}\right)-\left(\text{FP}\times \text{FN}\right)}{\sqrt{\left(\text{TP}+\text{FN}\right)\times \left(\text{TP}+\text{FP}\right)\times \left(\text{TN}+\text{FN}\right)\times \left(\text{TN}+\text{FP}\right)}} \end{equation*}

(17) \begin{equation*} \text{Precision}=\frac{\text{TP}}{\text{TP}+\text{FP}} \end{equation*}

(18) \begin{equation*} {F}_1=\frac{2\ast \text{Precision}\ast \text{Recall}}{\text{Precision}+\text{Recall}} \end{equation*}

where true positive (TP), true negative (TN), false positive (FP) and false negative (FN) denote the numbers of correctly identified positive samples, correctly identified negative samples, incorrectly identified positive samples and incorrectly identified negative samples, respectively. We also calculated the AUC in addition to these metrics.

## RESULT AND DISCUSSION

### Sequence pattern analysis

**Figure 2:**
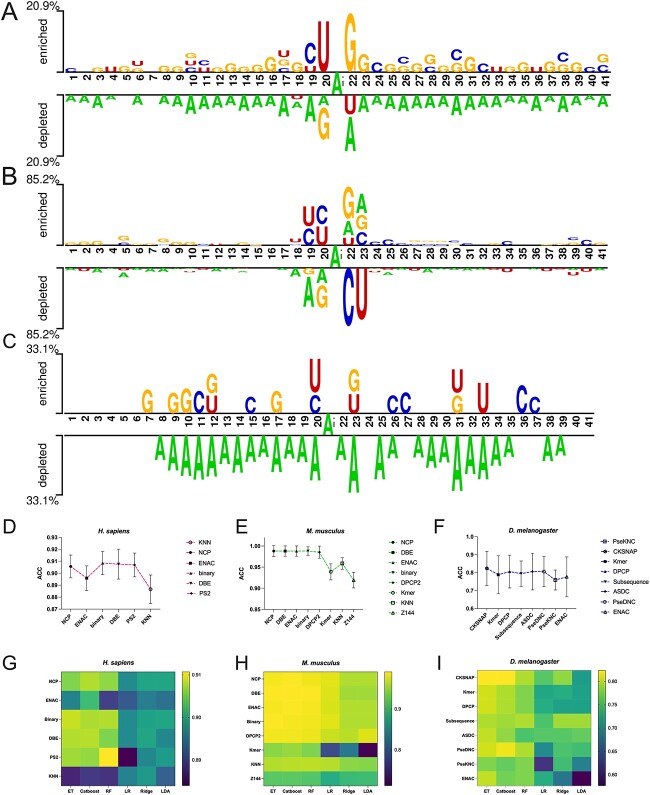
The analysis of RNA sequences, encoding methods and ML algorithms. Panels (**A**), (**B**) and (**C**) present the sequence logos of RNAs for *H. sapiens*, *M. musculus* and *D. melanogaster*, respectively, with positive samples up and negative ones down. Panels (**D**), (**E**) and (**F**) demonstrate 10-fold CV test results of the ET classifier trained with different encoding schemes on H_3000, M_831 and D_125 datasets, respectively. Panels (**G**), (**H**) and (**I**) show 10-fold CV test results of six base classifiers on H_3000, M_831 and D_125 datasets, respectively.

To better understand the sequence patterns of the RNA A-to-I editing sites, we analysed the patterns of the sequences in our dataset using Two sample logo [69], which is a web-based tool that detects and displays statistically significant differences in position-specific symbol compositions between two groups of sequences [69]. Figure 2A–C shows the base preferences for *H. sapiens*, *M. musculus* and *D. melanogaster*, respectively. The top panels represent positive samples, and the bottom panels represent negative samples. It can be concluded that the sequence patterns show different preferences around the adenine (A) sites at the centre. For *H. sapiens*, at position 27, guanine (G) is the most prevalent base, followed by U and C. In terms of the sequence pattern for *M. musculus*, U and C are the two bases that frequently occur, while A and G are the most frequent downstream bases. At the same time, the sequence logo of *D. melanogaster* demonstrates that RNA sequences are enriched in G and C at most nearby positions.

### Comparison of different feature encodings and base classifiers

To build a robust model, we comprehensively evaluated 37 RNA encoding schemes based on 10-fold CV results. We employed the ET [61] algorithm to conduct the experiments due to its relatively good predictive performance, extensive usage and rapid training time [42, 70, 71]. Consequently, we selected six to eight optimal feature encodings for each species based on the performance of ET. Based on the optimal feature encoding schemes selected by ET for each species, we then measured the prediction ability of 14 popular ML algorithms with respect to different species. To guarantee both the variety of classifiers and prediction ability for all three species, we ultimately selected three linear-based models, including LR, Ridge and LDA; and three tree-based models, including ET, Catboost and RF, as the base classifiers. Subsequently, we performed parameter optimization (e.g. *k*mer size and sliding window size) for the selected RNA encoding schemes to determine the optimal parameters for each encoding and built 132 models (22 encodings × 6 classifiers) accordingly. The optimal parameters of each feature are provided in Supplementary Table S1 available online at http://bib.oxfordjournals.org/, and the performance comparison results of these 132 models are provided in Supplementary Tables S2–S7 available online at http://bib.oxfordjournals.org/. Figure 2D–F shows the 10-fold CV test results of ET classifiers trained with different encoding schemes and Figure 2G–I shows the 10-fold CV test results of six base classifiers on H_3000, M_831 and D_125 datasets, respectively. For *H. sapiens*, nucleic acid composition-based encodings (NCP) and residue composition-based encodings (i.e. binary, DBE and PS2) can provide better discriminative ability than KNN and ENAC. In addition, these four encodings (NCP, binary, DBE and PS2) show almost the same contributions to the prediction performance. Therefore, we selected these four encodings for *H. sapiens* models for further analysis. In terms of *M. musculus*, nucleic acid composition-based encoding methods (NCP and ENAC), physicochemical property-based features (DPCP2) and residue composition-based features (DBE and binary) performed significantly better than others. Interestingly, we also observed the same phenomenon in *D. melanogaster*, where nucleic acid composition-based encodings (CKSNAP, Kmer and ASDC) performed exceptionally well compared with others. Physicochemical property-based features (DPCP) combined with pseudo nucleic acid composition-based features (PseDNC) achieved comparable performance and significantly higher accuracy than PseKNC and ENAC. Overall, nucleic acid composition-based encodings performed well among all three species. While physicochemical property-based and residue composition-based features have a high discriminative capability, they were much less effective than nucleic acid composition-based encodings. There are two possible reasons: (i) nucleic acid composition-based features provide information about the underlying genetic codes and can capture the inherent sequence-specific characteristics of A-to-I RNA editing sites; (ii) physicochemical property-based features extract indirect representations of the sequence using physicochemical properties of nucleotides, which may not be able to fully capture the underlying sequence-specific properties. Similarly, residue composition-based features consider only the occurrence frequencies of individual nucleotides in a sequence, failing to consider the ordering or arrangement of these residues in the local sequence context.

The aforementioned results demonstrate the value of examining numerous feature information across three benchmark datasets. In addition, the heatmap compares different base classifiers for each species, with colour indicating the level of accuracy. These performance comparison results reveal that tree-based classifiers performed better than linear-based classifiers across three species. Notably, Catboost and ET performed reasonably well in all three datasets; however, Ridge was marginally superior in *D. melanogaster*. In summary, CKSNAP is an optimal encoding for the *H. sapiens* dataset, while NCP is optimal for both the *M. musculus* and *D. melanogaster* datasets.

### Performance comparison of different ensemble strategies

**Figure 3:**
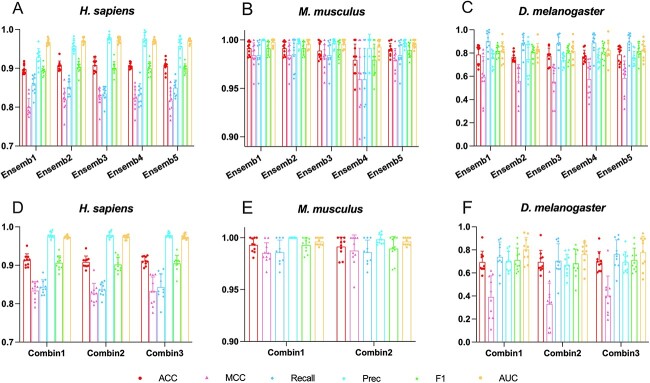
Performance comparison of different combinations of classifiers and encodings. Panels (**A**)–(**C**) show the performance comparison results of five ensemble strategies for the three species; panels (**D**)–(**F**) provide the corresponding comparisons of diverse encoding combinations.

To identify the optimal base classifiers from the six candidates to build the ensemble model, we assessed five different combinations of the base classifiers for each species using the 10-fold CV on the benchmark datasets. Given that stacking is time consuming, we adopted the optimal encoding for each species selected in Comparison of different feature encodings and base classifiers section to build the stacked ensemble models. The design of different ensemble methods follows the general principles: the two classifiers with good prediction accuracy are preferred for stacking, and the meta-model is selected accordingly to determine the strategy that provides the best predictive performance. In addition, considering that linear classifiers are not superior in general, we appropriately added linear classifiers for stacking to observe whether there are improvements in the overall prediction ability. Figure 3A–C and Supplementary Table S8 available online at http://bib.oxfordjournals.org/ represent the performance evaluation results of five different stacked models trained on three species-specific datasets. Regarding *H. sapiens*, despite relatively lower performance in recall and F_1_, the results revealed that Ensemble3 secured higher prediction performance in terms of ACC, MCC, Precision and AUC. Consequently, we selected Ensemble3 for *H. sapiens*, which used ET and RF as the base classifiers and employed ET as the meta-classifier. In terms of *M. musculus*, it should be noted that the model performance did not improve as the number of base classifiers increased. Ensemble1 and Ensemble2 achieved higher prediction performance (an accuracy of 0.9914) than the other three ensemble models. As shown in Figure 3B, Ensemble1 was superior to Ensemble2 due to its better Precision and AUC. Besides, we found that incorporating the linear model does not increase the prediction performance; therefore, we employed CatBoost and ET as the base classifiers and ET as the meta-classifier (Ensemble1) to build the stacked model. Owing to the smaller training dataset of *D. melanogaster* compared to the other two species, we employed more base classifiers to build stacked ensemble models to enhance the prediction accuracy and attempted to balance the number of tree-based and linear-based base classifiers. To serve these purposes, Ensemble5 is more suitable than Ensemble1. Besides, Figure 3C suggests that Ensemble5 secured the best predictive performance in terms of ACC (0.7882), MCC (0.5862), Precision (0.7570) and AUC (0.8236), together with a lower variance compared to Ensemble1. In summary, we applied Ensemble3 for *H. sapiens*, Ensemble1 for *M. musculus* and Ensemble5 for *D. melanogaster* in the following experiments*.*

### Performance comparison of different feature combinations

In this section, we optimised the stacked ensemble models selected in the Performance comparison of different ensemble strategies section for each species using a wide range of feature encoding combinations. Figure 3D–F and Supplementary Table S9 available online at http://bib.oxfordjournals.org/ show the 10-fold CV test results of different encoding combinations on the benchmark datasets. To ensure an objective comparison, we performed all the 10-fold CV tests based on the same partition of the training datasets. As diverse features are highly likely to provide more valuable information to enhance the effectiveness of models, we removed the features that are obviously inferior to their counterparts shown in the Comparison of different feature encodings and base classifiers section for different species and tested the predictive performance by incorporating the features from different groups. For *H. sapiens*, the new feature combination Combin1 achieved the best performance in all performance metrics except for AUC, with ACC of 0.9124, MCC of 0.8331, Recall of 0.8435, Precision of 0.9788 and F_1_ score of 0.9058. For *M. musculus*, we only examined two encoding combinations and slightly altered the settings of DPCP2, as the results in Figure 2E demonstrate that the performance of these five coding schemes did not vary significantly. As shown in Figure 3E, Combin1 is the superior encoding approach for *M. musculus*, evidenced by its outstanding predictive performance across all metrics. In terms of *D. melanogaster*, the ACC, MCC, Recall and F_1_ scores were achieved at 0.7000, 0.4015, 0.7611 and 0.7219, respectively, by Combin5, which outperformed the other two combinations. This is consistent with our previous findings in the Comparison of different feature encodings and base classifiers section that CKSNAP, Kmer, ASDC, PseDNC and DPCP are the best-performing encoding schemes. We ultimately selected Combin1 for *H. sapiens* and *M. musculus*, and Combin3 for *D. melanogaster*, respectively.

### Performance comparison among different feature selection strategies

**Figure 4:**
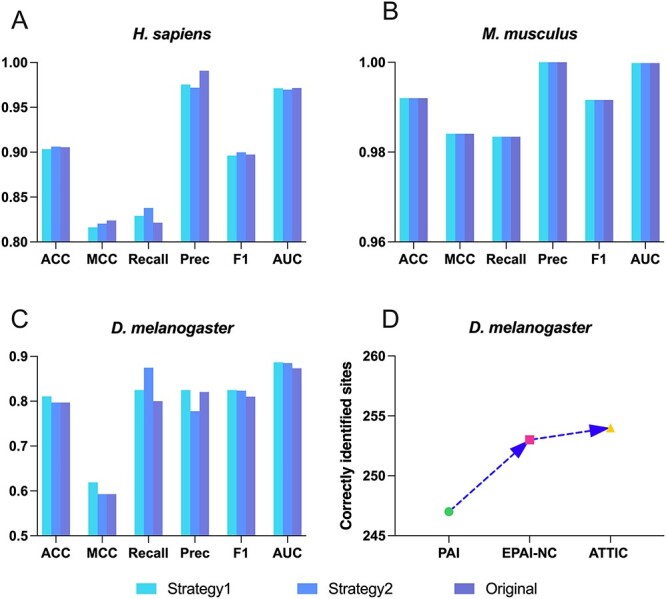
The performance evaluation results. Panels (**A**), (**B**) and (**C**) provide the comparison results of two different feature selection strategies in terms of ACC, MCC, Recall, Precision, F_1_ and AUC for *H. sapiens*, *M. musculus* and *D. melanogaster*. (**D**) Comparison with existing tools on the independent test datasets.

We trained the stacked ensemble models for each species according to the optimal ensemble strategies and feature combinations selected in the previous sections. However, the initial feature sets are likely to have some redundant and noisy features, which may have a negative impact on model performance. Therefore, we aimed to conduct feature selection to uncover informative feature subsets to enhance the prediction performance of stacked ensemble models. To this end, we compared two types of two-step feature selection strategies to determine the optimal feature subset for each species-specific model. Figure 4A–C illustrates the hold-out results of two feature selection strategies among three species. The number of selected features by each feature selection strategy and the corresponding prediction performance are provided in Supplementary Table S10 and Supplementary Figure S1 available online at http://bib.oxfordjournals.org/. As shown in Figure 4A, Strategy2 achieved the best prediction performance in terms of ACC, Recall and F_1_ score for *H. sapiens*, although the initial feature set got the best MCC, AUC and Precision. As we aim to improve prediction accuracy and reduce unnecessary features, Strategy2 was selected to optimize the *H. sapiens* model. As illustrated in Figure 4B, neither of the feature selection strategies enhanced the prediction performance on the M_831 dataset. However, given that the number of features selected by Strategy1 (i.e. 163) is smaller than that by Strategy2 (i.e. 322), we adopted Strategy1 to accelerate the model construction and avoid overfitting. In comparison, for *D. melanogaster*, it is worth noting that both feature selection strategies are helpful for improving prediction performance on the D_125 dataset, and Strategy1 outperformed Strategy2 in all assessment metrics except for Recall, with ACC of 0.8180, MCC of 0.6191, Precision of 0.8250, F_1_ score of 0.8250 and AUC of 0.8868, respectively. Therefore, Strategy1 was selected for *D. melanogaster* model optimisation.

### ATTIC outperformed state-of-the-art approaches for A-to-I editing site prediction

The final ATTIC framework was developed upon the optimal models improved by the feature selection strategy for specific species. We benchmarked ATTIC with several state-of-the-art approaches for different species: PAI [21], PAI-SAE [36] and iAI-DSAE [37] based on the D_125 dataset for *D. melanogaster*; iRNA-Ai [22] and iMRM [26] based on the H_3000 dataset for *H. sapiens*; and iRNA-3typeA [23] based on the M_831 dataset for *M. musculus*. We evaluated the performance of these models using the leave-one-out strategy, which was used in these methods. The ATTIC model of each species was built using the optimized features, base classifiers, feature selection approaches and stacking strategies concluded from our previous sections. Table 4 shows that ATTIC constantly outperformed the benchmarked methods regarding ACC, MCC, Recall and Sp scores regardless of species. Specifically, ATTIC outperformed other methods regarding all performance measures for A-to-I editing site prediction in *D. melanogaster*. Furthermore, ATTIC achieved satisfactory performance on the M_831 dataset. Except for Sp (0.9952), the other three measures improved significantly compared to iRNA-3typeA with ACC of 0.9946, MCC of 0.9892 and Recall of 0.9940. For all three species, we additionally provided AUC for further explanation. In addition, we assessed ATTIC and the other two methods, including PAI [21] and EPAI-NC [24], on the independent dataset (D_300). Figure 4D and Supplementary Table S11 available online at http://bib.oxfordjournals.org/ show the close prediction accuracy compared to the leave-one-out test results and indicate that ATTIC outperformed EPAI-NC and PAI in predicting A-to-I editing sites for *D. melanogaster*. Furthermore, to assess the generalization of all three models, we also conducted the independent tests by randomly selecting 30% of the samples as independent data and using the remaining 70% to train the model. The results of the independent test are provided in Supplementary Table S12 available online at http://bib.oxfordjournals.org/. We can see that the performance results are similar to those on LOOCV, suggesting that ATTIC has a good generalization ability. In conclusion, ATTIC shows superior predictive performance across three species, and the improved prediction performance enables a more accurate prediction in A-to-I RNA editing sites.

**Table 4:** Comparison with state-of-the-art methods based on the benchmark dataset

Species	Tool	ACC	MCC	Recall	Sp	AUC
*H. sapiens*	iRNA-Ai	0.9071	0.8200	0.8618	0.9523	–
	iMRM	0.9157	0.8310	0.8733	**0.9580**	–
	**ATTIC**	**0.9173**	**0.8363**	**0.8860**	0.9487	0.9749
*M. musculus*	iRNA-3typeA	0.9838	0.9600	0.9675	**1.0000**	–
	**ATTIC**	**0.9946**	**0.9892**	**0.9940**	0.9952	0.9990
*D. melanogaster*	PAI	0.7951	0.6000	0.8560	0.7311	–
	PAI-SAE	0.8197	0.6414	0.8720	0.7647	–
	iAI-DSAE	0.8197	0.6414	0.8720	0.7647	–
	**ATTIC**	**0.8360**	**0.6735**	**0.8800**	**0.7899**	**0.8726**

The bold values in the table represent the best performance of the corresponding metrics.

### Improving the model interpretation of ATTIC

**Figure 5:**
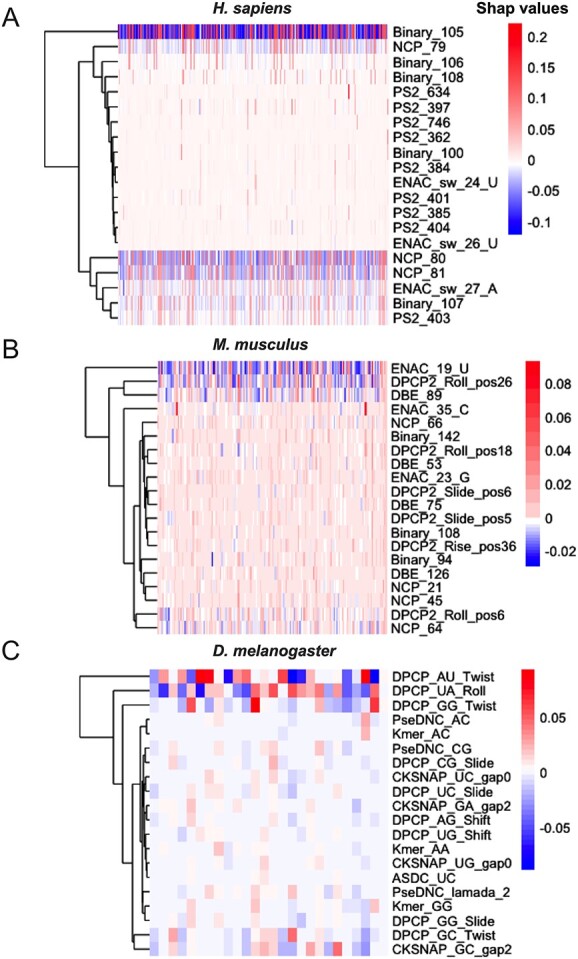
Top 20 features of ATTIC ranked by the SHAP algorithm for (**A**) *H. sapiens*, (**B**) *M. musculus* and (**C**) *D. melanogaster*. Each row represents a feature, and each column represents a sample. The colour represents the range of SHAP values, where the redder colour indicates that the SHAP value is greater than zero, and the bluer colour indicates that the SHAP value is below zero. Besides, the SHAP value greater than 0 indicates that the prediction tends to be a positive sample (i.e. A-to-I RNA editing site). In contrast, a SHAP value of less than 0 indicates that the prediction is likely to be a negative sample (i.e. non-A-to-I RNA editing site).

We applied the SHAP algorithm to interpret the feature importance to explain the outstanding prediction performance and robustness of the ATTIC model [72, 73]. Specifically, we employed the kernel SHAP, which can interpret the complicated stacked structure of ATTIC. The kernel SHAP estimates the SHAP values using a weighted local linear regression model [74]. Figure 5 shows the top 20 features ranked by the mean SHAP values integrated with all the testing samples. We noticed that nine PS2 features (PS2_634, PS2_397, PS2_746, PS2_362, PS2_384, PS2_401, PS2_385, PS2_404 and PS2_403) and five binary features (Binary_105, Binary_106, Binary_108, Binary_100 and Binary_107) are in the top 20 important features of *H. sapiens*, which are all residue composition-based features. In addition, most of these features motivate the positive A-to-I RNA editing prediction, while a few of the features (i.e. Binary_105, NCP_80 and NCP_81) both motivate positive and negative prediction. Likewise, residue composition-based features (binary and DBE) contribute significantly to predicting A-to-I sites in *M. musculus*. Apart from that, six physicochemical property-based features (DPCP2_Roll_pos26, DPCP2_Roll_pos18, DPCP2_Slide_pos6, DPCP2_Slide_pos5, DPCP2_Rise_pos36 and DPCP2_Roll_pos6) are crucial for ATTIC prediction either, especially with the roll property in the DPCP2. Regarding the prediction performance of ATTIC in *D. melanogaster*, DPCP accounts for approximately half of the top 20 important features. In addition, we found that the features containing GG and GC significantly contribute to the prediction, such as ‘DPCP_GG_Twist’, ‘PseDNC_CG’, ‘DPCP_CG_Slide’, ‘DPCP_GC_Twist’ and ‘CKSNAP_GC_gap2’. This is consistent with previous sequence pattern analyses that G and C are enriched in *D. melanogaster* RNAs in Sequence pattern analysis section. In short, these findings imply that residue composition-based properties and physicochemical property-based properties led to the improved prediction performance of ATTIC. To determine whether the top 20 features chosen by SHAP can distinguish A-to-I RNA editing in three species, we further explored the correlation values between the SHAP values of the top 20 features and the sample labels for each species. For all three species, the correlation ranged from 0.7818 to 0.9025 (*H. sapiens* is 0.7818, *M. musculus* is 0.8708, and *D. melanogaster* is 0.9035), indicating that these top 20 features selected by SHAP can identify A-to-I RNA editing effectively.

### Development of web server

To facilitate the community-wide usage of ATTIC for identifying potential A-to-I RNA editing sites, we implemented an online web server and a local stand-alone software package of the developed ATTIC approach. The web server is publicly available at https://web.unimelb-bioinfortools.cloud.edu.au/ATTIC/, while the stand-alone software package can be downloaded from GitHub (https://github.com/Cassie818/ATTIC). The web server of ATTIC is maintained with an Apache HTTP server configured on a 16-core Ubuntu server equipped with 32GB RAM and 500GB hard disk supported by the Research Cloud of The University of Melbourne. The web server requires a few steps to predict the potential A-to-I RNA editing sites for a given RNA sequence. First, users are required to input or upload the query RNA sequences in the FASTA format. Next, users need to specify the species of interest and the corresponding threshold. Finally, the job will be submitted to the server by clicking the ‘Submit’ button. After the job is finished, the generated results will be returned to the webpage or emailed to the user’s email address if provided. The results webpage lists the query sequence, predicted probabilities, species types and predicted results (A-to-I editing site or not). In addition, the help webpage of the web server provides detailed guidance for users to understand and interpret the results.

### Limitations and future work

Despite the promising performance of ATTIC in predicting A-to-I RNA editing sites across the three different species, it has the following limitations.

The first major limitation is that the training dataset used in ATTIC is relatively limited. To ensure that the dataset used in this study is consistent in distribution and that a stable and robust machine learning model could be trained, we only utilized the data retrieved from the DARNED database. In future work, we plan to develop deep learning-based methods to enable the integration of multiple datasets from different databases, such as RADAR and REDIportal [4, 14, 17], to explore the possibility of further enhancing the predictive performance.

The second major limitation is that ATTIC is unable to predict tissue-specific A-to-I RNA editing sites. Previous studies have shown that A-to-I RNA editing can significantly vary across cells, tissues and developmental stages, thereby affecting multiple different aspects of RNA metabolism [75]. Accordingly, it would be of particular value to develop tissue-specific predictors based on the tissue-specific RNA editing knowledge annotated by RNA editing databases such as RADAR and REDIportal [4, 14, 17] in future work.

## CONCLUSION

In this study, we proposed ATTIC, a novel stacking-based predictor for identifying A-to-I RNA editing sites in *H. sapiens*, *M. musculus* and *D. melanogaster*. We first comprehensively evaluated the prediction performance of 14 widely established ML models with 37 types of RNA sequence features and then built a series of stacked ensemble models for optimisation. We then built the final ATTIC model for each species upon the optimal feature combinations, stacking strategies and feature selection approaches. The LOO and independent test results demonstrate that ATTIC outperformed state-of-the-art tools for predicting A-to-I editing sites in three species. Besides, we employed the SHAP algorithm to analyse the contributions of important features to the superior performance of ATTIC. Finally, we developed a user-friendly web server and software package to facilitate community use of ATTIC. To the best of our knowledge, ATTIC is the first stacking-based predictor that enables the detection of A-to-I RNA sites in *H. sapiens*, *M. musculus* and *D. melanogaster*. The success of ATTIC can be attributed to the following factors: (i) the comprehensive selection of the optimal ensemble strategies and feature combinations for each species; (ii) using feature selection approaches to effectively remove the redundant features. Although ATTIC significantly increased the prediction accuracy of *M. musculus* and *D. melanogaster*, the improvement for *H. sapiens* is limited. Besides, we trained three models independently that lack generalizability to other species. We anticipate that ATTIC can be utilised as a useful tool for predicting A-to-I RNA editing sites and improving our understanding of their roles in transcriptional modification.

**Key Points**

- Accurate identification of A-to-I editing sites is crucial for understanding RNA-level modifications and their functional roles in regulating cellular processes.
- We proposed a novel stacked predictor, termed ATTIC, to identify A-to-I editing sites across three species, including *Homo sapiens*, *Mus musculus* and *Drosophila melanogaster*.
- Extensive cross-validation and independent tests illustrate that ATTIC outperforms state-of-the-art tools for predicting A-to-I editing sites.
- The online web server and local stand-alone tool of ATTIC are freely available at http://web.unimelb-bioinfortools.cloud.edu.au/ATTIC/ for the wider research community to use.

## Supplementary Material

Supplementary_Data_final_bbad170

## Data Availability

The source code and datasets of ATTIC are publicly available at http://web.unimelb-bioinfortools.cloud.edu.au/ATTIC/.

## Author Biographies

**Ruyi Chen** is currently a PhD student at The University of Queensland. Her research interests are bioinformatics and machine learning.

**Fuyi Li** is currently a professor at the College of Information Engineering, Northwest A&F University, China. He is also affliated with the Peter Doherty Institute for Infection and Immunity, The University of Melbourne. His research interests are bioinformatics, computational biology, machine learning and data mining.

**Xudong Guo** received his MEng degree from Ningxia University, China. He is currently a research assistant at the College of Information Engineering, Northwest A&F University, China. His research interests are bioinformatics and data mining.

**Yue Bi** is currently a PhD student in the Department of Biochemistry and Molecular Biology and Biomedicine Discovery Institute, Monash University, Australia. Her research interests include bioinformatics, computational biology, sequence analysis and machine learning.

**Chen Li** is a Research Fellow at the Biomedicine Discovery Institute and Department of Biochemistry and Molecular Biology, Monash University. His research interests include systems proteomics, immunopeptidomes, personalized medicine and experimental bioinformatics.

**Shirui Pan** is a Professor and an ARC Future Fellow with the School of Information and Communication Technology, Griffith University, Australia. His research interests are machine learning and data mining.

**Lachlan J.M. Coin** is a professor and group leader in the Department of Microbiology and Immunology at the University of Melbourne. He is also a member of the Department of Clinical Pathology, University of Melbourne. His research interests are bioinformatics, machine learning, transcriptomics and genomics.

**Jiangning Song** is an associate professor and a group leader in the Monash Biomedicine Discovery Institute, Monash University. He is also affiliated with the Monash Data Futures Institute, Monash University. His research interests include bioinformatics, computational biomedicine, machine learning, data mining and pattern recognition.

## References

[1] Mallela A, Nishikura K. A-to-I editing of protein coding and noncoding RNAs. Crit Rev Biochem Mol Biol 2012;47:493–501. 22988838 10.3109/10409238.2012.714350

[2] Gray MW . Evolutionary origin of RNA editing. Biochemistry 2012;51:5235–42. 22708551 10.1021/bi300419r

[3] Nishikura K . Functions and regulation of RNA editing by ADAR deaminases. Annu Rev Biochem 2010;79:321–49. 20192758 10.1146/annurev-biochem-060208-105251 PMC2953425

[4] Ramaswami G, Li JB. RADAR: a rigorously annotated database of A-to-I RNA editing. Nucleic Acids Res 2014;42:D109–13. 24163250 10.1093/nar/gkt996 PMC3965033

[5] Amariglio N, Rechavi G. A-to-I RNA editing: a new regulatory mechanism of global gene expression. Blood Cells Mol Dis 2007;39:151–5. 17555993 10.1016/j.bcmd.2007.04.003

[6] Zhang Z, Carmichael GG. The fate of dsRNA in the nucleus. Cell 2001;106:465–76. 11525732 10.1016/s0092-8674(01)00466-4

[7] Ivanov A, Memczak S, Wyler E, et al. Analysis of intron sequences reveals hallmarks of circular RNA biogenesis in animals. Cell Rep 2015;10:170–7. 25558066 10.1016/j.celrep.2014.12.019

[8] Maas S, Kawahara Y, Tamburro KM, et al. A-to-I RNA editing and human disease. RNA Biol 2006;3:1–9. 17114938 10.4161/rna.3.1.2495 PMC2947206

[9] Nigita G, Veneziano D, Ferro A. A-to-I RNA editing: current knowledge sources and computational approaches with special emphasis on non-coding RNA molecules. Front Bioeng Biotechnol 2015;3:37. 10.3389/fbioe.2015.00037 PMC4373398 25859542

[10] Han L, Diao L, Yu S, et al. The genomic landscape and clinical relevance of A-to-I RNA editing in human cancers. Cancer Cell 2015;28:515–28. 26439496 10.1016/j.ccell.2015.08.013 PMC4605878

[11] Ishizuka JJ, Manguso RT, Cheruiyot CK, et al. Loss of ADAR1 in tumours overcomes resistance to immune checkpoint blockade. Nature 2019;565:43–8. 30559380 10.1038/s41586-018-0768-9 PMC7241251

[12] Liu H, Golji J, Brodeur LK, et al. Tumor-derived IFN triggers chronic pathway agonism and sensitivity to ADAR loss. Nat Med 2019;25:95–102. 30559422 10.1038/s41591-018-0302-5

[13] Kiran A, Baranov PV. DARNED: a DAtabase of RNa EDiting in humans. Bioinformatics 2010;26:1772–6. 20547637 10.1093/bioinformatics/btq285

[14] Mansi L, Tangaro MA, Lo Giudice C, et al. REDIportal: millions of novel A-to-I RNA editing events from thousands of RNAseq experiments. Nucleic Acids Res 2021;49:D1012–9. 33104797 10.1093/nar/gkaa916 PMC7778987

[15] Alon S, Garrett SC, Levanon EY, et al. The majority of transcripts in the squid nervous system are extensively recoded by A-to-I RNA editing. Elife 2015;4:e05198. 25569156 10.7554/eLife.05198 PMC4384741

[16] Li JB, Levanon EY, Yoon J-K, et al. Genome-wide identification of human RNA editing sites by parallel DNA capturing and sequencing. Science 2009;324:1210–3. 19478186 10.1126/science.1170995

[17] Picardi E, D’Erchia AM, Lo Giudice C, et al. REDIportal: a comprehensive database of A-to-I RNA editing events in humans. Nucleic Acids Res 2017;45:D750–7. 27587585 10.1093/nar/gkw767 PMC5210607

[18] Li M, Wang IX, Li Y, et al. Widespread RNA and DNA sequence differences in the human transcriptome. Science 2011;333:53–8. 21596952 10.1126/science.1207018 PMC3204392

[19] Kim E, Magen A, Ast G. Different levels of alternative splicing among eukaryotes. Nucleic Acids Res 2007;35:125–31. 17158149 10.1093/nar/gkl924 PMC1802581

[20] Lo Giudice C, Tangaro MA, Pesole G, et al. Investigating RNA editing in deep transcriptome datasets with REDItools and REDIportal. Nat Protoc 2020;15:1098–131. 31996844 10.1038/s41596-019-0279-7

[21] Chen W, Feng P, Ding H, et al. PAI: predicting adenosine to inosine editing sites by using pseudo nucleotide compositions. Sci Rep 2016;6:35123. 27725762 10.1038/srep35123 PMC5057124

[22] Chen W, Feng P, Yang H, et al. iRNA-AI: identifying the adenosine to inosine editing sites in RNA sequences. Oncotarget 2017;8:4208–17. 27926534 10.18632/oncotarget.13758 PMC5354824

[23] Chen W, Feng P, Yang H, et al. iRNA-3typeA: identifying three types of modification at RNA’s adenosine sites. Mol Ther Nucleic Acids 2018;11:468–74. 29858081 10.1016/j.omtn.2018.03.012 PMC5992483

[24] Ahmad A, Shatabda S. EPAI-NC: enhanced prediction of adenosine to inosine RNA editing sites using nucleotide compositions. Anal Biochem 2019;569:16–21. 30664849 10.1016/j.ab.2019.01.002

[25] Choyon A, Rahman A, Hasanuzzaman M, et al. PRESa2i: incremental decision trees for prediction of adenosine to inosine RNA editing sites. F1000Res 2020;9:262.

[26] Liu K, Chen W. iMRM: a platform for simultaneously identifying multiple kinds of RNA modifications. Bioinformatics 2020;36:3336–42. 32134472 10.1093/bioinformatics/btaa155

[27] Kim M, Hur B, Kim S. RDDpred: a condition-specific RNA-editing prediction model from RNA-seq data. BMC Genomics 2016;17(Suppl 1):5. 26817607 10.1186/s12864-015-2301-y PMC4895604

[28] Xiong H, Liu D, Li Q, et al. RED-ML: a novel, effective RNA editing detection method based on machine learning. GigaScience 2017;6:gix012. 10.1093/gigascience/gix012 PMC5467039 28328004

[29] Ouyang Z, Liu F, Zhao C, et al. Accurate identification of RNA editing sites from primitive sequence with deep neural networks. Sci Rep 2018;8:6005. 29662087 10.1038/s41598-018-24298-y PMC5902551

[30] Tongnueasuk P, Wichadakul D. TAE-ML: a random forest model for detecting RNA editing sites. In: 2020 17th International Joint Conference on Computer Science and Software Engineering (JCSSE). IEEE, 2020; 59–64

[31] Tac HA, Koroglu M, Sezerman U. RDDSVM: accurate prediction of A-to-I RNA editing sites from sequence using support vector machines. Funct Integr Genomics 2021;21:633–43. 34529170 10.1007/s10142-021-00805-9

[32] Wang J, Ness S, Brown R, et al. EditPredict: prediction of RNA editable sites with convolutional neural network. Genomics 2021;113:3864–71. 34562567 10.1016/j.ygeno.2021.09.016 PMC8671215

[33] Peng Z, Cheng Y, Tan BC-M, et al. Comprehensive analysis of RNA-Seq data reveals extensive RNA editing in a human transcriptome. Nat Biotechnol 2012;30:253–60. 22327324 10.1038/nbt.2122

[34] Pinto Y, Cohen HY, Levanon EY. Mammalian conserved ADAR targets comprise only a small fragment of the human editosome. Genome Biol 2014;15:1–15. 10.1186/gb-2014-15-1-r5 PMC4053846 24393560

[35] Chen L-L, Carmichael GG. Altered nuclear retention of mRNAs containing inverted repeats in human embryonic stem cells: functional role of a nuclear noncoding RNA. Mol Cell 2009;35:467–78. 19716791 10.1016/j.molcel.2009.06.027 PMC2749223

[36] Xiao X, Wang P, Xu Z, et al. PAI-SAE: predicting adenosine to inosine editing sites based on hybrid features by using spare auto-encoder. IOP Conf Ser: Earth Environ Sci 2018;170:052018. 10.1088/1755-1315/170/5/052018.

[37] Xu Z-C, Xiao X, Qiu W-R, et al. iAI-DSAE: a computational method for adenosine to inosine editing site prediction. LOC 2019;16:347–55.

[38] St Laurent G, Tackett MR, Nechkin S, et al. Genome-wide analysis of A-to-I RNA editing by single-molecule sequencing in Drosophila. Nat Struct Mol Biol 2013;20:1333–9. 24077224 10.1038/nsmb.2675

[39] Song Z, Huang D, Song B, et al. Attention-based multi-label neural networks for integrated prediction and interpretation of twelve widely occurring RNA modifications. Nat Commun 2021;12:4011. 34188054 10.1038/s41467-021-24313-3 PMC8242015

[40] Fu L, Niu B, Zhu Z, et al. CD-HIT: accelerated for clustering the next-generation sequencing data. Bioinformatics 2012;28:3150–2. 23060610 10.1093/bioinformatics/bts565 PMC3516142

[41] Yu Y, Zhou H, Kong Y, et al. The landscape of A-to-I RNA Editome is shaped by both positive and purifying selection. PLoS Genet 2016;12:e1006191. 27467689 10.1371/journal.pgen.1006191 PMC4965139

[42] Chen Z, Liu X, Zhao P, et al. *iFeatureOmega*: an integrative platform for engineering, visualization and analysis of features from molecular sequences, structural and ligand data sets. Nucleic Acids Res 2022;50:W434–47. 35524557 10.1093/nar/gkac351 PMC9252729

[43] Lee D, Karchin R, Beer MA. Discriminative prediction of mammalian enhancers from DNA sequence. Genome Res 2011;21:2167–80. 21875935 10.1101/gr.121905.111 PMC3227105

[44] Zhang Z-Y, Yang Y-H, Ding H, et al. Design powerful predictor for mRNA subcellular location prediction in *Homo sapiens*. Brief Bioinform 2021;22:526–35. 31994694 10.1093/bib/bbz177

[45] Chen Z, Zhao P, Li F, et al. iLearn: an integrated platform and meta-learner for feature engineering, machine-learning analysis and modeling of DNA, RNA and protein sequence data. Brief Bioinform 2020;21:1047–57. 31067315 10.1093/bib/bbz041

[46] Wei L, Zhou C, Chen H, et al. ACPred-FL: a sequence-based predictor using effective feature representation to improve the prediction of anti-cancer peptides. Bioinformatics 2018;34:4007–16. 29868903 10.1093/bioinformatics/bty451 PMC6247924

[47] Chen W, Tran H, Liang Z, et al. Identification and analysis of the N6-methyladenosine in the Saccharomyces cerevisiae transcriptome. Sci Rep 2015;5:13859. 26343792 10.1038/srep13859 PMC4561376

[48] Zheng L, Huang S, Mu N, et al. RAACBook: a web server of reduced amino acid alphabet for sequence-dependent inference by using Chou’s five-step rule. Database 2019;2019:baz131. 31802128 10.1093/database/baz131 PMC6893003

[49] Chen W, Feng P-M, Lin H, et al. iRSpot-PseDNC: identify recombination spots with pseudo dinucleotide composition. Nucleic Acids Res 2013;41:e68–8. 23303794 10.1093/nar/gks1450 PMC3616736

[50] Qiang X, Chen H, Ye X, et al. M6AMRFS: robust prediction of N6-methyladenosine sites with sequence-based features in multiple species. Front Genet 2018;9:495. 30410501 10.3389/fgene.2018.00495 PMC6209681

[51] Doench JG, Fusi N, Sullender M, et al. Optimized sgRNA design to maximize activity and minimize off-target effects of CRISPR-Cas9. Nat Biotechnol 2016;34:184–91. 26780180 10.1038/nbt.3437 PMC4744125

[52] Li J, Huang Y, Yang X, et al. RNAm5Cfinder: a web-server for predicting RNA 5-methylcytosine (m5C) sites based on random forest. Sci Rep 2018;8:17299. 30470762 10.1038/s41598-018-35502-4 PMC6251864

[53] Zhang S, Shi H. iR5hmcSC: identifying RNA 5-hydroxymethylcytosine with multiple features based on stacking learning. Comput Biol Chem 2021;95:107583. 34562726 10.1016/j.compbiolchem.2021.107583

[54] Liu B, Gao X, Zhang H. BioSeq-Analysis2.0: an updated platform for analyzing DNA, RNA and protein sequences at sequence level and residue level based on machine learning approaches. Nucleic Acids Res 2019;47:e127–7. 31504851 10.1093/nar/gkz740 PMC6847461

[55] Mishra A, Pokhrel P, Hoque MT. StackDPPred: a stacking based prediction of DNA-binding protein from sequence. Bioinformatics 2019;35:433–41. 30032213 10.1093/bioinformatics/bty653

[56] Wei L, He W, Malik A, et al. Computational prediction and interpretation of cell-specific replication origin sites from multiple eukaryotes by exploiting stacking framework. Brief Bioinform 2021;22:bbaa275. 33152766 10.1093/bib/bbaa275

[57] Fu X, Cai L, Zeng X, et al. StackCPPred: a stacking and pairwise energy content-based prediction of cell-penetrating peptides and their uptake efficiency. Bioinformatics 2020;36:3028–34. 32105326 10.1093/bioinformatics/btaa131

[58] Panta M, Mishra A, Hoque MT, et al. ClassifyTE: a stacking-based prediction of hierarchical classification of transposable elements. Bioinformatics 2021;37:2529–36. 33682878 10.1093/bioinformatics/btab146

[59] Basith S, Lee G, Manavalan B. STALLION: a stacking-based ensemble learning framework for prokaryotic lysine acetylation site prediction. Brief Bioinform 2022;23:bbab376. 34532736 10.1093/bib/bbab376 PMC8769686

[60] Dorogush AV, Ershov V, Gulin A. CatBoost: gradient boosting with categorical features support, arXiv:1810.11363, 2018.

[61] Geurts P, Ernst D, Wehenkel L. Extremely randomized trees. Mach Learn 2006;63:3–42.

[62] Friedman JH . Greedy function approximation: a gradient boosting machine. Ann Stat 2001;29:1189–232.

[63] Ke G, Meng Q, Finley T, et al. LightGBM: a highly efficient gradient boosting decision tree. Adv Neural Inf Process Syst 2017;30:3149–57.

[64] Pedregosa F, Varoquaux G, Gramfort A, et al. Scikit-learn: machine learning in python. J Mach Learn Res 2011;12:2825–30.

[65] Ferri FJ, Pudil P, Hatef M, et al. Comparative study of techniques for large-scale feature selection. Mach Intell Pattern Recogn 1994;16:403–13.

[66] Rodriguez-Galiano VF, Luque-Espinar JA, Chica-Olmo M, et al. Feature selection approaches for predictive modelling of groundwater nitrate pollution: an evaluation of filters, embedded and wrapper methods. Sci Total Environ 2018;624:661–72. 29272835 10.1016/j.scitotenv.2017.12.152

[67] Zeng X, Zhang X, Zou Q. Integrative approaches for predicting microRNA function and prioritizing disease-related microRNA using biological interaction networks. Brief Bioinform 2016;17:193–3. 26059461 10.1093/bib/bbv033

[68] Gao S, Wang P, Feng Y, et al. RIFS2D: a two-dimensional version of a randomly restarted incremental feature selection algorithm with an application for detecting low-ranked biomarkers. Comput Biol Med 2021;133:104405. 33930763 10.1016/j.compbiomed.2021.104405

[69] Vacic V, Iakoucheva LM, Radivojac P. Two sample logo: a graphical representation of the differences between two sets of sequence alignments. Bioinformatics 2006;22:1536–7. 16632492 10.1093/bioinformatics/btl151

[70] Shafique R, Mehmood A, Ullah S, et al. Cardiovascular disease prediction system using extra trees classifier. 2019. 10.21203/rs.2.14454/v1.

[71] Abhishek L. Optical character recognition using ensemble of SVM, MLP and extra trees classifier. In: 2020 International Conference for Emerging Technology (INCET) 2020; 1–4

[72] Lundberg SM, Erion GG, Lee S-I. Consistent Individualized Feature Attribution for Tree Ensembles, arXiv:1802.03888, 2018.

[73] Marcilio WE, Eler DM. From explanations to feature selection: assessing SHAP values as feature selection mechanism. In: 2020 33rd SIBGRAPI Conference on Graphics, Patterns and Images (SIBGRAPI). IEEE, 2020; 340–7

[74] Lundberg SM, Lee S-I. A unified approach to interpreting model predictions. Adv Neural Inf Process Syst 2017;30:4768–77.

[75] GTEx Consortium, Tan MH, Li Q, et al. Dynamic landscape and regulation of RNA editing in mammals. Nature 2017;550:249–54. 29022589 10.1038/nature24041 PMC5723435

