# Supplementary material for: ATTIC is an integrated approach for predicting A-to-I RNA editing sites in three species
Source: Brief Bioinform. 2023 May 6;24(3):bbad170. doi: 10.1093/bib/bbad170 (PMC10565902; doi:10.1093/bib/bbad170)
Supplement: Supplementary_Data_final_bbad170 [file supplementary_data_final_bbad170.docx]

**Supplementary Data**

Table of Contents

[Table S1. The parameters of different encoding schemes of iFeatureOmega. 9](#_Toc130282641)

[Table S2. The 10-fold CV results of the ET classifier trained using different encoding methods and datasets. 10](#_Toc130282642)

[Table S3. The 10-fold CV results of the Catboost classifier trained using different encoding methods and datasets. 12](#_Toc130282643)

[Table S4. The 10-fold CV results of the RF classifier trained using different encoding methods and datasets. 14](#_Toc130282644)

[Table S5. The 10-fold CV results of the LR classifier on different encoding methods and datasets. 16](#_Toc130282645)

[Table S6. The 10-fold CV results of the Ridge classifier on different encoding methods and datasets. 18](#_Toc130282646)

[Table S7. The 10-fold CV results of the LDA classifier on different encoding methods and datasets. 20](#_Toc130282647)

[Table S8. The l0-fold CV results of different ensemble methods. 22](#_Toc130282648)

[Table S9. The 10-fold CV results of different encoding combinations. 24](#_Toc130282649)

[Table S10. The comparison of two incremental feature selection methods across three species. 25](#_Toc130282650)

[Table S11. Performance comparison with the existing tools on the independent test dataset. 26](#_Toc130282651)

[Table S12. Performance evaluation results of ATTIC on *H. sapiens*, *M. musculus* and *D. melanogaster* datasets 26](#_Toc130282652)

[Fig S1. The IFS curves of three benchmark datasets using two incremental feature selection methods. (A) *D. melanogaster*; (B) *M.* *musculus*; (C) *H. sapiens.* 27](#_Toc130282653)

**Feature engineering**

**KNN encoding (KNN)**

K-nearest neighbours for peptides (KNN) require an additional training file and a label file. The training file is used to determine the best KNN nucleotides acid by computing the similarity score between two peptide sequences [1].

**The occurrences of kmers allowing non-contiguous matches (Subsequence)**

The subsequence descriptor allows a non-contiguous matching [2], for instance, the 3-mer “GCA” in the RNA sequence “GCAUCG”. There are four kinds of possibilities by means of exact and non-contiguous matching, including “GCA”, “-GCA”, “G-CA”, and “GC-A”, where “-” indicates the gap in non-contiguous matching. In this case, “GCA” is the exact form, while “-GCA”, “G-CA”, and “GC-A” are non-contiguous forms. The frequency of non-contiguous forms is penalised with their length *L* and the factor δ (0 < δ < 1), defined as δ*^L^*. Thereby, the occurrence of “GCA” is $1 + 2\delta^{6}+ \delta^{5}$ [3].

**The Z curve parameters for frequencies of phase-specific tri-nucleotides (Z144)**

The frequencies of the bases “A”, “C”, “G”, and “U” occurring in an open reading frame or a piece of RNA sequence with bases at positions 1, 4, 7, ...; 2, 5, 8, ...; 3, 6, 9, ... are represented as a_1_, c_1_, g_1_, t_1_; a_2_, c_2_, g_2_, t_2_; a_3_, c_3_, g_3_, t_3_, separately. They are the base frequencies at the first, second, and third codon locations. a_i_, c_i_, g_i_, and t_i_ are mapped onto a point P_i_ in a three-dimensional space V_i_, i=1, 2, 3. The Z-transform of DNA sequences determines the *P_i_* coordinates, indicated as *x_i_*, *y_i_*, and *z_i_* [3,4]. The Z144 descriptor can be calculated as follows:

$$\left\{ \begin{aligned} x_{XY}^{k} = (p^{k}(XYA) + p^{k}(XYG))-(p^{k}(XYC) + p^{k}(XYU)), \\ y_{XY}^{k} = (p^{k}(XYA) + p^{k}(XYC))-(p^{k}(XYG) + p^{k}(XYU)), \\ z_{XY}^{k} = (p^{k}(XYA) + p^{k}(XYU))-(p^{k}(XYG) + p^{k}(XYC)), \end{aligned} \right.$$

where X = A, C, G, U; Y = A, C, G, U; k = 1,2,3.

**To facilitate the readers’ understanding of the defined features, we provided several toy examples for all the features used in ATTIC as below:**

NCP:


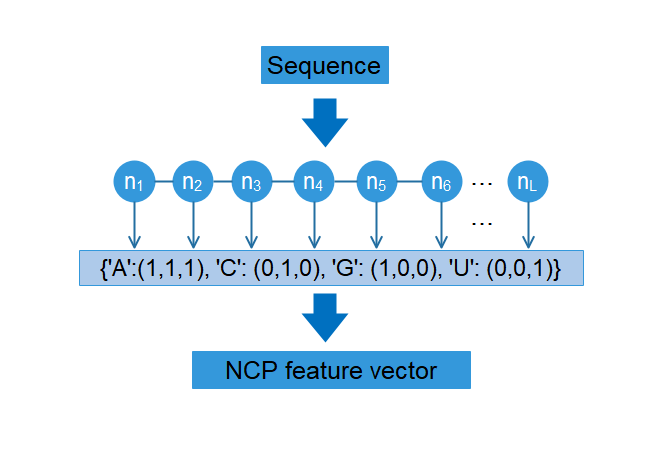


ENAC:


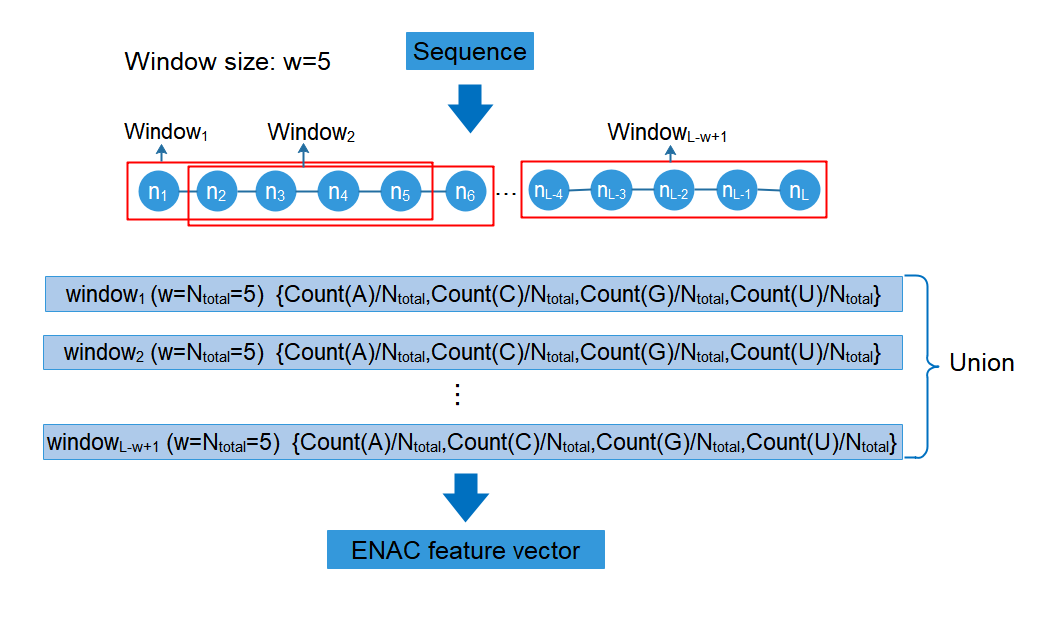


Binary:


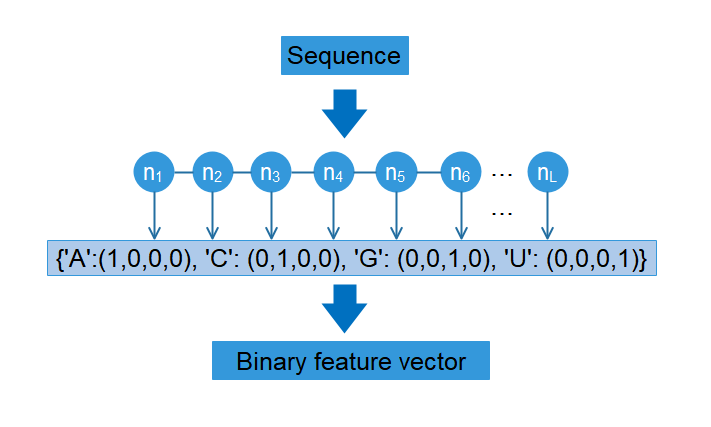


DBE:


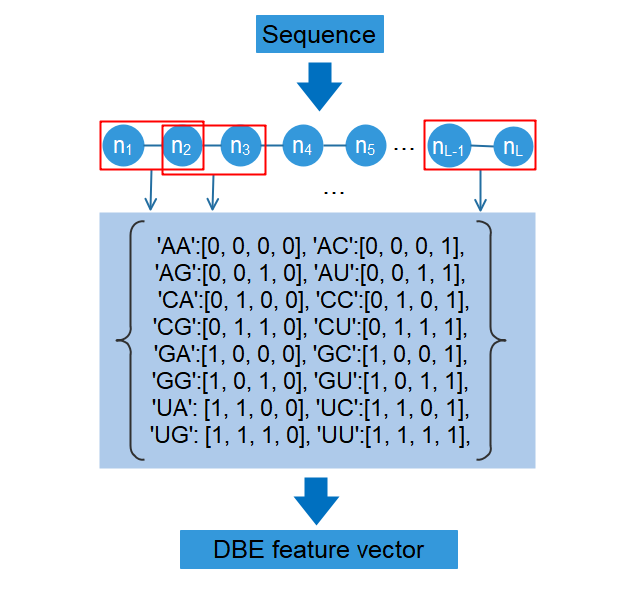


PS2:


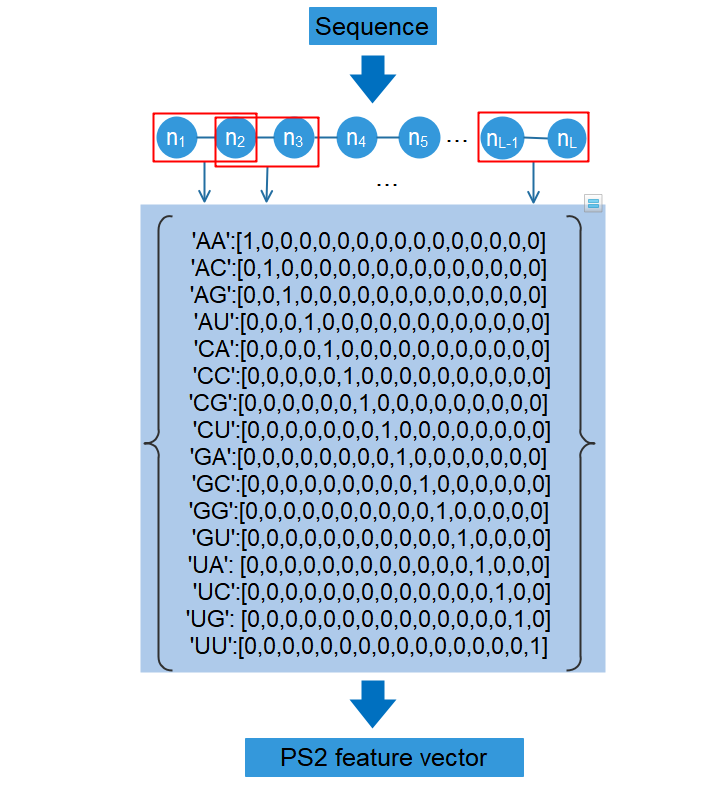


DPCP:


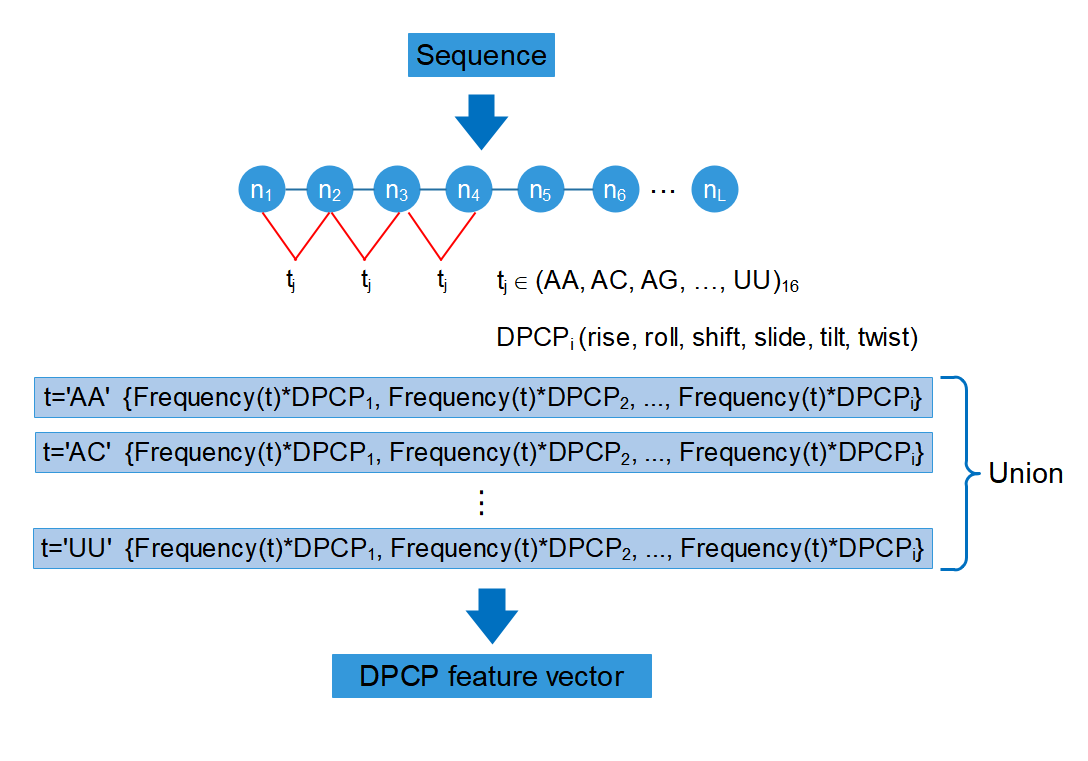


DPCP2:


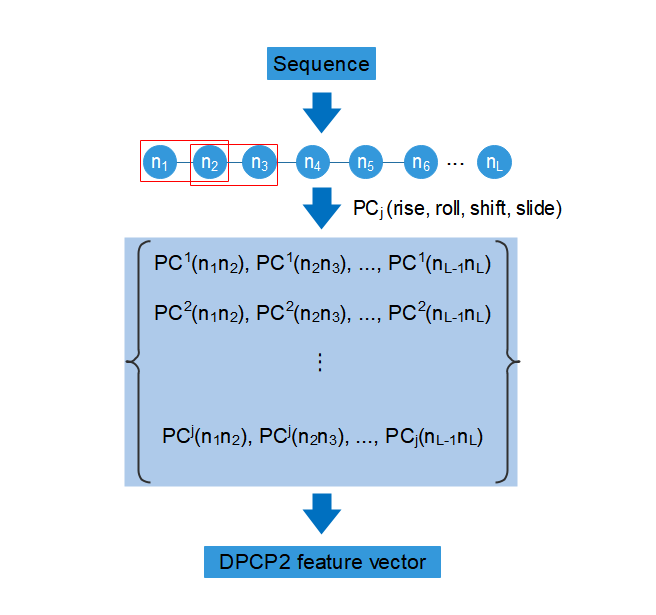


Kmer:


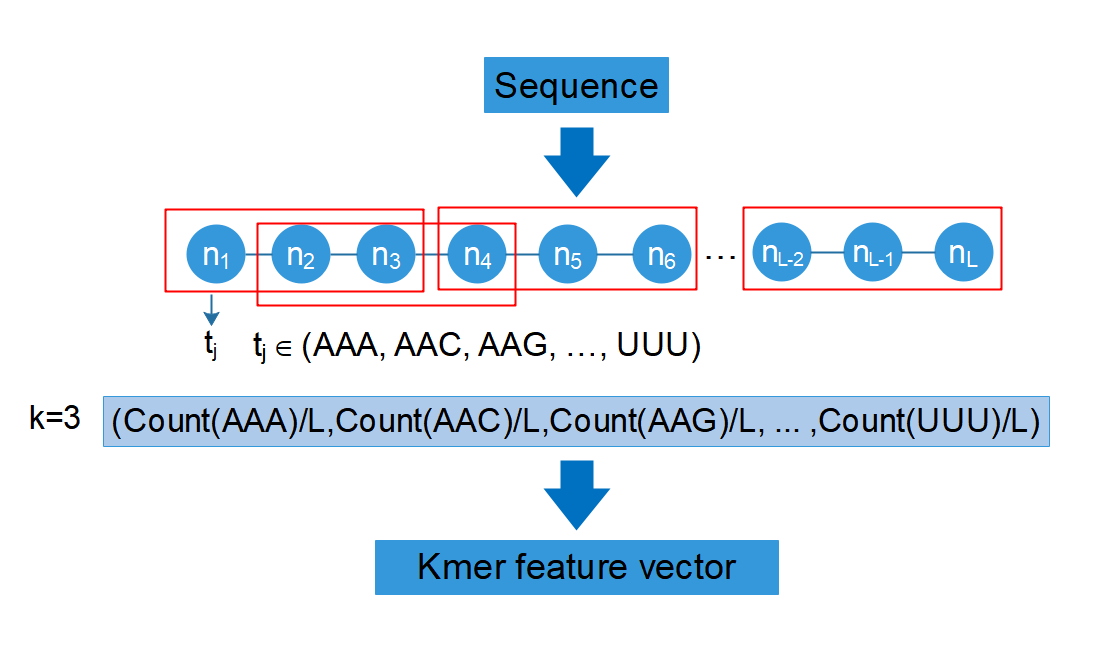


CKSNAP:


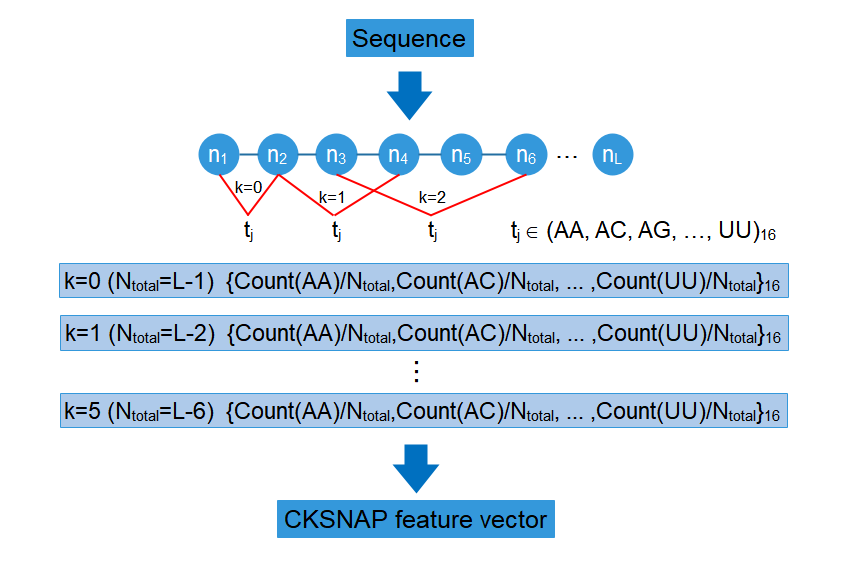


PseDNC:


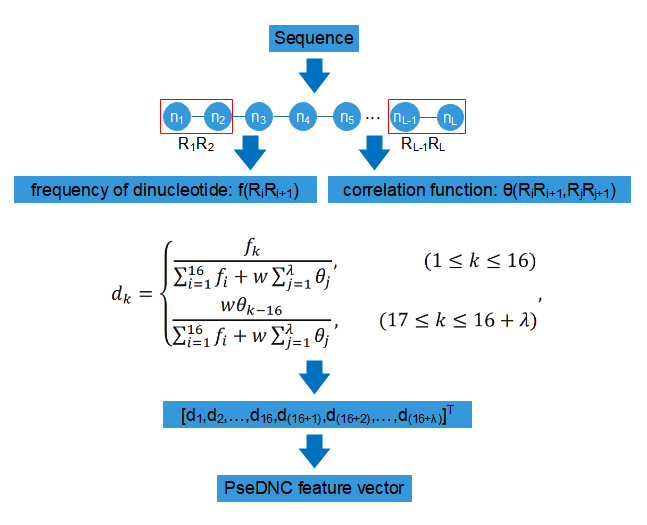


**Machine learning algorithms**

Catboost employs gradient boosting on decision trees to create a series of decision trees during the training process, with the subsequent trees producing less loss compared to the preceding ones [5]. RF utilises DT as the fundamental unit and is a bagging-based ensemble learning algorithm for solving classification problems [6,7]. ET is also an ensemble method based on unpruned regression or decision trees and differs from other tree-based ensemble models. ET separates nodes depending on the randomly generated cut-points, and it does not use bootstrap replica but the total learned samples to create the trees [8]. As one of the linear classifiers, LR is a typical probabilistic classification model that examines the connection between several independent variables and a categorical dependent variable and calculates the likelihood of an event occurring by fitting the data to a logistic curve [9,10]. Ridge is an efficient approach that provides a regularisation hyperparameter to conventional multiple linear regression for analysing multicollinear data [11]. By selecting the projection hyperplane, LDA minimises the interclass variance and maximises the projected distance between class means. It can be achieved by solving an eigenvalue problem where the matched eigenvector defines the desired hyperplane that can be used for the classification [12].

## Table S1. The parameters of different encoding schemes of iFeatureOmega.

| **Species** | **Encoding schemes** | **Parameters** |
| --- | --- | --- |
| H_3000 | NCP | default |
|  | ENAC | Sliding window size = 2 |
|  | binary | default |
|  | DBE | default |
|  | PS2 | default |
|  | KNN | default |
| M_831 | NCP | default |
|  | DBE | default |
|  | binary | default |
|  | KNN | default |
|  | ENAC | Sliding window size = 2 |
|  | DPCP2 | Rise, Roll, Shift, Slide |
|  | Kmer | Kmer size = 6 |
| D_125 | CKSNAP | Kmer size = 2 |
|  | Kmer | Kmer size = 2 |
|  | DPCP | Rise, Roll, Shift, Slide, Tilt, Twist |
|  | Subsequence | *k* = 3, delta = 1 |
|  | ASDC | default |
|  | PseDNC | lambda = 6; Rise, Roll, Shift, Slide, Tilt, Twist |
|  | PseKNC | Kmer size = 5; Rise, Roll, Shift, Slide, Tilt, Twist |
|  | ENAC | Sliding window size = 8 |

## Table S2. The 10-fold CV results of the ET classifier trained using different encoding methods and datasets.

| **Dataset** | **Encoding scheme** | **ACC** | **AUC** | **Recall** | **Precision** | **F1** | **Kappa** | **MCC** |
| --- | --- | --- | --- | --- | --- | --- | --- | --- |
| D_125 | CKSNAP | 0.8235±0.0949 | 0.8702±0.0903 | 0.9144±0.1235 | 0.8023±0.0925 | 0.8479±0.0828 | 0.6405±0.1927 | 0.6643±0.1847 |
|  | Kmer | 0.8059±0.0986 | 0.8737±0.1064 | 0.8933±0.1047 | 0.7909±0.0996 | 0.8342±0.0830 | 0.6037±0.2016 | 0.6216±0.1948 |
|  | DPCP | 0.8059±0.0913 | 0.8721±0.0899 | 0.8611±0.1321 | 0.8121±0.0990 | 0.8264±0.0827 | 0.6075±0.1842 | 0.6258±0.1781 |
|  | Subsequence | 0.7941±0.0708 | 0.8508±0.1005 | 0.8356±0.0884 | 0.7971±0.0735 | 0.8126±0.0631 | 0.5839±0.1442 | 0.5907±0.1452 |
|  | ASDC | 0.8000±0.1294 | 0.8594±0.0921 | 0.8522±0.1603 | 0.8016±0.1094 | 0.8196±0.1208 | 0.5973±0.2579 | 0.6092±0.2584 |
|  | PseDNC | 0.8059±0.0834 | 0.8869±0.0783 | 0.8611±0.1160 | 0.8072±0.0825 | 0.8270±0.0725 | 0.6069±0.1706 | 0.6211±0.1668 |
|  | PseKNC | 0.7765±0.0735 | 0.8453±0.0997 | 0.8056±0.1607 | 0.8011±0.0925 | 0.7895±0.0832 | 0.5504±0.1469 | 0.5733±0.1498 |
|  | ENAC | 0.8000±0.0956 | 0.8500±0.0874 | 0.8822±0.1348 | 0.7889±0.0879 | 0.8246±0.0852 | 0.5951±0.1938 | 0.6184±0.1745 |
| M_831 | NCP | 0.9871±0.0116 | 0.9980±0.0025 | 0.9739±0.0235 | 1.0000±0.0000 | 0.9866±0.0123 | 0.9742±0.0233 | 0.9748±0.0224 |
|  | DBE | 0.9906±0.0124 | 0.9979±0.0024 | 0.9809±0.0249 | 1.0000±0.0000 | 0.9902±0.0130 | 0.9811±0.0247 | 0.9816±0.0238 |
|  | ENAC | 0.9871±0.0116 | 0.9991±0.0013 | 0.9739±0.0235 | 1.0000±0.0000 | 0.9866±0.0123 | 0.9742±0.0233 | 0.9748±0.0224 |
|  | binary | 0.9897±0.0120 | 0.9970±0.0035 | 0.9792±0.0241 | 1.0000±0.0000 | 0.9893±0.0126 | 0.9794±0.0240 | 0.9799±0.0230 |
|  | DPCP2 | 0.9854±0.0143 | 0.9977±0.0036 | 0.9705±0.0289 | 1.0000±0.0000 | 0.9848±0.0152 | 0.9708±0.0287 | 0.9716±0.0274 |
|  | Kmer | 0.9424±0.0187 | 0.9862±0.0071 | 0.9216±0.0261 | 0.9609±0.0278 | 0.9405±0.0191 | 0.8848±0.0375 | 0.8861±0.0376 |
|  | KNN | 0.9587±0.0138 | 0.9900±0.0060 | 0.9425±0.0270 | 0.9736±0.0209 | 0.9574±0.0145 | 0.9174±0.0276 | 0.9185±0.0276 |
|  | Z144 | 0.9192±0.0184 | 0.9672±0.0178 | 0.8799±0.0339 | 0.9535±0.0228 | 0.9147±0.0197 | 0.8382±0.0369 | 0.8414±0.0364 |
| H_3000 | NCP | 0.9057±0.0095 | 0.9690±0.0046 | 0.8394±0.0132 | 0.9714±0.0117 | 0.9005±0.0102 | 0.8118±0.0190 | 0.8197±0.0188 |
|  | ENAC | 0.8959±0.0103 | 0.9602±0.0072 | 0.8235±0.0159 | 0.9671±0.0119 | 0.8894±0.0115 | 0.7923±0.0206 | 0.8016±0.0201 |
|  | binary | 0.9085±0.0096 | 0.9697±0.0032 | 0.8408±0.0147 | 0.9761±0.0085 | 0.9034±0.0106 | 0.8175±0.0191 | 0.8258±0.0184 |
|  | DBE | 0.9076±0.0125 | 0.9690±0.0059 | 0.8441±0.0232 | 0.9706±0.0119 | 0.9027±0.0141 | 0.8156±0.0248 | 0.8230±0.0232 |
|  | PS2 | 0.9069±0.0099 | 0.9700±0.0039 | 0.8488±0.0158 | 0.9640±0.0131 | 0.9026±0.0107 | 0.8141±0.0196 | 0.8203±0.0194 |
|  | KNN | 0.8866±0.0119 | 0.9550±0.0067 | 0.8544±0.0082 | 0.9175±0.0202 | 0.8847±0.0112 | 0.7735±0.0239 | 0.7756±0.0248 |

## Table S3. The 10-fold CV results of the Catboost classifier trained using different encoding methods and datasets.

| **Dataset** | **Encoding scheme** | **ACC** | **AUC** | **Recall** | **Precision** | **F1** | **Kappa** | **MCC** |
| --- | --- | --- | --- | --- | --- | --- | --- | --- |
| D_125 | CKSNAP | 0.8235±0.1019 | 0.8646±0.1239 | 0.9367±0.1056 | 0.7970±0.1079 | 0.8542±0.0812 | 0.6376±0.2101 | 0.6704±0.1873 |
|  | Kmer | 0.7941±0.1124 | 0.8562±0.1049 | 0.8611±0.1224 | 0.8004±0.1190 | 0.8206±0.0921 | 0.5822±0.2302 | 0.5987±0.2252 |
|  | DPCP | 0.7941±0.1124 | 0.8617±0.0942 | 0.8611±0.1224 | 0.7961±0.1113 | 0.8203±0.0937 | 0.5817±0.2294 | 0.5958±0.2261 |
|  | Subsequence | 0.7882±0.0956 | 0.8521±0.1175 | 0.8467±0.1006 | 0.7930±0.0992 | 0.8122±0.0720 | 0.5701±0.1990 | 0.5818±0.2020 |
|  | ASDC | 0.7824±0.1207 | 0.8323±0.1085 | 0.8611±0.1497 | 0.7741±0.1086 | 0.8090±0.1126 | 0.5572±0.2438 | 0.5742±0.2398 |
|  | PseDNC | 0.8176±0.0928 | 0.8449±0.1250 | 0.8922±0.0670 | 0.8077±0.0951 | 0.8440±0.0681 | 0.6277±0.1938 | 0.6377±0.1860 |
|  | PseKNC | 0.7647±0.0832 | 0.8312±0.0879 | 0.8589±0.0989 | 0.7637±0.1186 | 0.7988±0.0661 | 0.5200±0.1721 | 0.5446±0.1684 |
|  | ENAC | 0.7706±0.1246 | 0.8464±0.0789 | 0.8500±0.1533 | 0.7664±0.1074 | 0.7972±0.1127 | 0.5372±0.2509 | 0.5521±0.2456 |
| M_831 | NCP | 0.9897±0.0100 | 0.9963±0.0046 | 0.9792±0.0201 | 1.0000±0.0000 | 0.9894±0.0104 | 0.9794±0.0200 | 0.9798±0.0194 |
|  | DBE | 0.9905±0.0105 | 0.9962±0.0053 | 0.9809±0.0211 | 1.0000±0.0000 | 0.9902±0.0109 | 0.9811±0.0209 | 0.9815±0.0203 |
|  | ENAC | 0.9897±0.0100 | 0.9966±0.0048 | 0.9809±0.0196 | 0.9982±0.0053 | 0.9894±0.0104 | 0.9794±0.0200 | 0.9797±0.0194 |
|  | binary | 0.9871±0.0128 | 0.9968±0.0041 | 0.9757±0.0247 | 0.9982±0.0054 | 0.9867±0.0135 | 0.9742±0.0257 | 0.9747±0.0249 |
|  | DPCP2 | 0.9854±0.0121 | 0.9964±0.0048 | 0.9704±0.0245 | 1.0000±0.0000 | 0.9848±0.0128 | 0.9708±0.0243 | 0.9714±0.0234 |
|  | Kmer | 0.9476±0.0200 | 0.9826±0.0095 | 0.9304±0.0243 | 0.9629±0.0275 | 0.9461±0.0202 | 0.8951±0.0400 | 0.8961±0.0402 |
|  | KNN | 0.9622±0.0111 | 0.9919±0.0052 | 0.9459±0.0226 | 0.9773±0.0205 | 0.9610±0.0115 | 0.9243±0.0222 | 0.9253±0.0223 |
|  | Z144 | 0.9200±0.0208 | 0.9713±0.0135 | 0.8765±0.0452 | 0.9593±0.0288 | 0.9150±0.0231 | 0.8399±0.0417 | 0.8445±0.0403 |
| H_3000 | NCP | 0.9076±0.0099 | 0.9689±0.0048 | 0.8460±0.0166 | 0.9686±0.0131 | 0.9030±0.0108 | 0.8156±0.0196 | 0.8225±0.0192 |
|  | ENAC | 0.9024±0.0098 | 0.9673±0.0056 | 0.8492±0.0175 | 0.9540±0.0135 | 0.8984±0.0108 | 0.8050±0.0196 | 0.8103±0.0192 |
|  | binary | 0.9074±0.0063 | 0.9694±0.0046 | 0.8478±0.0149 | 0.9662±0.0126 | 0.9030±0.0070 | 0.8151±0.0125 | 0.8217±0.0122 |
|  | DBE | 0.9078±0.0097 | 0.9699±0.0057 | 0.8445±0.0197 | 0.9707±0.0124 | 0.9030±0.0110 | 0.8160±0.0194 | 0.8235±0.0181 |
|  | PS2 | 0.9064±0.0066 | 0.9691±0.0040 | 0.8530±0.0087 | 0.9587±0.0144 | 0.9027±0.0064 | 0.8131±0.0131 | 0.8184±0.0141 |
|  | KNN | 0.8885±0.0111 | 0.9567±0.0072 | 0.8596±0.0129 | 0.9164±0.0150 | 0.8870±0.0112 | 0.7773±0.0223 | 0.7789±0.0224 |

## Table S4. The 10-fold CV results of the RF classifier trained using different encoding methods and datasets.

| **Dataset** | **Encoding scheme** | **ACC** | **AUC** | **Recall** | **Precision** | **F1** | **Kappa** | **MCC** |
| --- | --- | --- | --- | --- | --- | --- | --- | --- |
| D_125 | CKSNAP | 0.7941±0.1213 | 0.8633±0.1373 | 0.8600±0.1319 | 0.8055±0.1397 | 0.8198±0.1002 | 0.5826±0.2483 | 0.6026±0.2489 |
|  | Kmer | 0.7765±0.0941 | 0.8588±0.1114 | 0.8722±0.1194 | 0.7622±0.0788 | 0.8079±0.0789 | 0.5451±0.1919 | 0.5621±0.1896 |
|  | DPCP | 0.7765±0.1110 | 0.8610±0.1020 | 0.8400±0.1283 | 0.7821±0.1079 | 0.8027±0.0989 | 0.5478±0.2227 | 0.5594±0.2169 |
|  | Subsequence | 0.7941±0.0882 | 0.8509±0.1159 | 0.8578±0.0850 | 0.7920±0.0901 | 0.8187±0.0679 | 0.5816±0.1840 | 0.5909±0.1771 |
|  | ASDC | 0.7529±0.1171 | 0.8178±0.1118 | 0.8278±0.1152 | 0.7618±0.1225 | 0.7862±0.0947 | 0.4965±0.2406 | 0.5116±0.2389 |
|  | PseDNC | 0.7941±0.1027 | 0.8401±0.1235 | 0.8600±0.0944 | 0.7999±0.1167 | 0.8218±0.0798 | 0.5810±0.2127 | 0.5957±0.2048 |
|  | PseKNC | 0.7765±0.0780 | 0.8634±0.0745 | 0.8478±0.1418 | 0.7741±0.0909 | 0.8004±0.0815 | 0.5461±0.1557 | 0.5714±0.1507 |
|  | ENAC | 0.7765±0.1046 | 0.8361±0.1008 | 0.8389±0.1541 | 0.7866±0.1132 | 0.7994±0.0992 | 0.5497±0.2087 | 0.5757±0.1936 |
| M_831 | NCP | 0.9845±0.0120 | 0.9963±0.0041 | 0.9687±0.0242 | 1.0000±0.0000 | 0.9839±0.0126 | 0.9690±0.0240 | 0.9698±0.0231 |
|  | DBE | 0.9897±0.0100 | 0.9963±0.0053 | 0.9792±0.0201 | 1.0000±0.0000 | 0.9894±0.0104 | 0.9794±0.0200 | 0.9798±0.0194 |
|  | ENAC | 0.9871±0.0110 | 0.9975±0.0035 | 0.9740±0.0222 | 1.0000±0.0000 | 0.9867±0.0114 | 0.9742±0.0220 | 0.9748±0.0214 |
|  | binary | 0.9854±0.0153 | 0.9980±0.0023 | 0.9705±0.0309 | 1.0000±0.0000 | 0.9848±0.0162 | 0.9708±0.0307 | 0.9716±0.0294 |
|  | DPCP2 | 0.9828±0.0153 | 0.9970±0.0036 | 0.9652±0.0309 | 1.0000±0.0000 | 0.9820±0.0163 | 0.9656±0.0307 | 0.9666±0.0293 |
|  | Kmer | 0.9372±0.0164 | 0.9824±0.0062 | 0.9164±0.0245 | 0.9553±0.0250 | 0.9351±0.0170 | 0.8744±0.0328 | 0.8756±0.0330 |
|  | KNN | 0.9630±0.0134 | 0.9909±0.0061 | 0.9476±0.0235 | 0.9772±0.0205 | 0.9619±0.0139 | 0.9260±0.0268 | 0.9269±0.0269 |
|  | Z144 | 0.9158±0.0190 | 0.9648±0.0147 | 0.8731±0.0459 | 0.9536±0.0237 | 0.9106±0.0215 | 0.8314±0.0380 | 0.8358±0.0359 |
| H_3000 | NCP | 0.9062±0.0122 | 0.9680±0.0039 | 0.8343±0.0225 | 0.9782±0.0106 | 0.9003±0.0141 | 0.8128±0.0243 | 0.8222±0.0224 |
|  | ENAC | 0.8890±0.0061 | 0.9590±0.0076 | 0.8076±0.0128 | 0.9692±0.0064 | 0.8809±0.0073 | 0.7786±0.0121 | 0.7901±0.0109 |
|  | binary | 0.9076±0.0075 | 0.9698±0.0041 | 0.8329±0.0156 | 0.9830±0.0095 | 0.9016±0.0086 | 0.8156±0.0149 | 0.8258±0.0138 |
|  | DBE | 0.9055±0.0075 | 0.9688±0.0047 | 0.8441±0.0160 | 0.9659±0.0096 | 0.9008±0.0086 | 0.8113±0.0150 | 0.8182±0.0140 |
|  | PS2 | 0.9107±0.0072 | 0.9696±0.0045 | 0.8516±0.0158 | 0.9694±0.0129 | 0.9065±0.0080 | 0.8217±0.0143 | 0.8283±0.0137 |
|  | KNN | 0.8878±0.0115 | 0.9557±0.0073 | 0.8549±0.0146 | 0.9192±0.0157 | 0.8858±0.0116 | 0.7759±0.0229 | 0.7780±0.0231 |

## Table S5. The 10-fold CV results of the LR classifier on different encoding methods and datasets.

| **Dataset** | **Encoding scheme** | **ACC** | **AUC** | **Recall** | **Precision** | **F1** | **Kappa** | **MCC** |
| --- | --- | --- | --- | --- | --- | --- | --- | --- |
| D_125 | CKSNAP | 0.7588±0.1034 | 0.8171±0.0885 | 0.8833±0.1192 | 0.7372±0.0900 | 0.7988±0.0846 | 0.5056±0.2139 | 0.5289±0.2159 |
|  | Kmer | 0.7235±0.0746 | 0.7920±0.0799 | 0.8833±0.1192 | 0.7005±0.0691 | 0.7747±0.0599 | 0.4313±0.1563 | 0.4698±0.1560 |
|  | DPCP | 0.7294±0.0798 | 0.8296±0.0876 | 0.7756±0.1223 | 0.7528±0.0931 | 0.7545±0.0702 | 0.4567±0.1623 | 0.4710±0.1647 |
|  | Subsequence | 0.7529±0.0824 | 0.7877±0.0644 | 0.7478±0.1205 | 0.7842±0.0950 | 0.7607±0.0941 | 0.5059±0.1624 | 0.5121±0.1644 |
|  | ASDC | 0.7588±0.0965 | 0.8144±0.0903 | 0.9144±0.1235 | 0.7249±0.0808 | 0.8029±0.0786 | 0.5045±0.1984 | 0.5427±0.1948 |
|  | PseDNC | 0.6941±0.0977 | 0.7961±0.0793 | 0.9578±0.0846 | 0.6551±0.0849 | 0.7735±0.0668 | 0.3607±0.2050 | 0.4373±0.1865 |
|  | PseKNC | 0.6118±0.0798 | 0.8240±0.0765 | 0.9889±0.0333 | 0.5871±0.0614 | 0.7349±0.0488 | 0.1715±0.1585 | 0.2458±0.1943 |
|  | ENAC | 0.7294±0.0880 | 0.7866±0.1043 | 0.7733±0.1387 | 0.7543±0.1182 | 0.7526±0.0854 | 0.4550±0.1782 | 0.4731±0.1849 |
| M_831 | NCP | 0.9785±0.0181 | 0.9933±0.0069 | 0.9600±0.0337 | 0.9964±0.0107 | 0.9776±0.0192 | 0.9570±0.0362 | 0.9581±0.0348 |
|  | DBE | 0.9759±0.0120 | 0.9940±0.0069 | 0.9600±0.0269 | 0.9913±0.0118 | 0.9751±0.0127 | 0.9518±0.0241 | 0.9528±0.0234 |
|  | ENAC | 0.9742±0.0158 | 0.9946±0.0067 | 0.9513±0.0307 | 0.9964±0.0072 | 0.9730±0.0169 | 0.9484±0.0316 | 0.9497±0.0301 |
|  | binary | 0.9785±0.0181 | 0.9933±0.0069 | 0.9600±0.0337 | 0.9964±0.0107 | 0.9776±0.0192 | 0.9570±0.0362 | 0.9581±0.0348 |
|  | DPCP2 | 0.9674±0.0183 | 0.9868±0.0107 | 0.9356±0.0387 | 0.9983±0.0052 | 0.9655±0.0199 | 0.9346±0.0366 | 0.9371±0.0344 |
|  | Kmer | 0.7764±0.0402 | 0.9310±0.0177 | 0.5605±0.0821 | 0.9764±0.0294 | 0.7085±0.0681 | 0.5499±0.0821 | 0.6073±0.0676 |
|  | KNN | 0.9596±0.0145 | 0.9928±0.0046 | 0.9407±0.0273 | 0.9771±0.0205 | 0.9582±0.0151 | 0.9191±0.0290 | 0.9203±0.0287 |
|  | Z144 | 0.8840±0.0247 | 0.9433±0.0254 | 0.8468±0.0393 | 0.9131±0.0352 | 0.8779±0.0263 | 0.7677±0.0495 | 0.7708±0.0506 |
| H_3000 | NCP | 0.8966±0.0083 | 0.9663±0.0043 | 0.8628±0.0154 | 0.9297±0.0212 | 0.8947±0.0078 | 0.7935±0.0167 | 0.7961±0.0176 |
|  | ENAC | 0.8914±0.0071 | 0.9597±0.0050 | 0.8642±0.0109 | 0.9179±0.0155 | 0.8901±0.0065 | 0.7830±0.0141 | 0.7846±0.0147 |
|  | binary | 0.8966±0.0083 | 0.9663±0.0043 | 0.8628±0.0145 | 0.9297±0.0212 | 0.8947±0.0078 | 0.7935±0.0167 | 0.7961±0.0176 |
|  | DBE | 0.8966±0.0082 | 0.9657±0.0055 | 0.8502±0.0190 | 0.9417±0.0209 | 0.8933±0.0084 | 0.7936±0.0164 | 0.7980±0.0169 |
|  | PS2 | 0.8840±0.0123 | 0.9522±0.0071 | 0.8792±0.0141 | 0.8917±0.0186 | 0.8853±0.0118 | 0.7680±0.0246 | 0.7684±0.0246 |
|  | KNN | 0.8957±0.0064 | 0.9591±0.0063 | 0.8769±0.0098 | 0.9147±0.0106 | 0.8953±0.0064 | 0.7915±0.0129 | 0.7923±0.0130 |

## Table S6. The 10-fold CV results of the Ridge classifier on different encoding methods and datasets.

| **Dataset** | **Encoding scheme** | **ACC** | **Recall** | **Precision** | **F1** | **Kappa** | **MCC** |
| --- | --- | --- | --- | --- | --- | --- | --- |
| D_125 | CKSNAP | 0.7824±0.0986 | 0.9033±0.1023 | 0.7548±0.0902 | 0.8185±0.0803 | 0.5536±0.2030 | 0.5746±0.2026 |
|  | Kmer | 0.7294±0.0840 | 0.8511±0.1145 | 0.7168±0.0785 | 0.7725±0.0693 | 0.4468±0.1742 | 0.4690±0.1776 |
|  | DPCP | 0.7176±0.0865 | 0.7644±0.1266 | 0.7387±0.0886 | 0.7436±0.0780 | 0.4325±0.1748 | 0.4446±0.1784 |
|  | Subsequence | 0.7882±0.0753 | 0.8467±0.0875 | 0.7853±0.0735 | 0.8112±0.0621 | 0.5695±0.1574 | 0.5781±0.1534 |
|  | ASDC | 0.7588±0.0965 | 0.8611±0.1321 | 0.7464±0.0852 | 0.7928±0.0837 | 0.5091±0.1960 | 0.5297±0.1959 |
|  | PseDNC | 0.7529±0.0904 | 0.9044±0.1202 | 0.7231±0.0837 | 0.7980±0.0754 | 0.4916±0.1865 | 0.5300±0.1824 |
|  | PseKNC | 0.7471±0.0746 | 0.9478±0.0849 | 0.7028±0.0648 | 0.8031±0.0516 | 0.4739±0.1589 | 0.5289±0.1438 |
|  | ENAC | 0.6706±0.0956 | 0.6989±0.1739 | 0.7178±0.1271 | 0.6860±0.1160 | 0.3432±0.1851 | 0.3612±0.1853 |
| M_831 | NCP | 0.9579±0.0231 | 0.9147±0.0466 | 1.0000±0.0000 | 0.9548±0.0258 | 0.9157±0.0463 | 0.9197±0.0425 |
|  | DBE | 0.9519±0.0210 | 0.9025±0.0423 | 1.0000±0.0000 | 0.9482±0.0241 | 0.9036±0.0420 | 0.9084±0.0378 |
|  | ENAC | 0.9588±0.0232 | 0.9165±0.0468 | 1.0000±0.0000 | 0.9558±0.0260 | 0.9174±0.0465 | 0.9214±0.0426 |
|  | binary | 0.9579±0.0231 | 0.9147±0.0466 | 1.0000±0.0000 | 0.9548±0.0258 | 0.9157±0.0463 | 0.9197±0.0425 |
|  | DPCP2 | 0.9622±0.0210 | 0.9234±0.0426 | 1.0000±0.0000 | 0.9597±0.0231 | 0.9243±0.0422 | 0.9277±0.0393 |
|  | Kmer | 0.8246±0.0280 | 0.6773±0.0490 | 0.9536±0.0296 | 0.7912±0.0378 | 0.6476±0.0568 | 0.6764±0.0532 |
|  | KNN | 0.9424±0.0192 | 0.8850±0.0374 | 0.9980±0.0060 | 0.9377±0.0219 | 0.8846±0.0385 | 0.8906±0.0352 |
|  | Z144 | 0.9012±0.0269 | 0.8764±0.0349 | 0.9201±0.0321 | 0.8974±0.0281 | 0.8022±0.0538 | 0.8037±0.0539 |
| H_3000 | NCP | 0.8997±0.0087 | 0.8057±0.0179 | 0.9966±0.0051 | 0.8909±0.0105 | 0.8001±0.0172 | 0.8157±0.0146 |
|  | ENAC | 0.8952±0.0081 | 0.8118±0.0174 | 0.9789±0.0134 | 0.8873±0.0094 | 0.7910±0.0160 | 0.8033±0.0150 |
|  | binary | 0.8997±0.0087 | 0.8057±0.0179 | 0.9966±0.0051 | 0.8909±0.0105 | 0.8001±0.0172 | 0.8157±0.0146 |
|  | DBE | 0.8964±0.0105 | 0.8085±0.0211 | 0.9854±0.0108 | 0.8880±0.0126 | 0.7934±0.0209 | 0.8071±0.0187 |
|  | PS2 | 0.8983±0.0100 | 0.8375±0.0192 | 0.9574±0.0130 | 0.8933±0.0112 | 0.7970±0.0198 | 0.8038±0.0190 |
|  | KNN | 0.8928±0.0060 | 0.8493±0.0119 | 0.9344±0.0131 | 0.8896±0.0062 | 0.7859±0.0120 | 0.7895±0.0124 |

## Table S7. The 10-fold CV results of the LDA classifier on different encoding methods and datasets.

| **Dataset** | **Encoding scheme** | **ACC** | **AUC** | **Recall** | **Precision** | **F1** | **Kappa** | **MCC** |
| --- | --- | --- | --- | --- | --- | --- | --- | --- |
| D_125 | CKSNAP | 0.7118±0.1327 | 0.7585±0.1000 | 0.7300±0.1737 | 0.7460±0.1159 | 0.7272±0.1275 | 0.4220±0.2660 | 0.4326±0.2690 |
|  | Kmer | 0.7176±0.0865 | 0.8183±0.0957 | 0.7644±0.1266 | 0.7387±0.0886 | 0.7436±0.0780 | 0.4325±0.1748 | 0.4446±0.1784 |
|  | DPCP | 0.7176±0.0865 | 0.8183±0.0957 | 0.7644±0.1266 | 0.7387±0.0886 | 0.7436±0.0780 | 0.4325±0.1748 | 0.4446±0.1784 |
|  | Subsequence | 0.7882±0.0753 | 0.7940±0.1171 | 0.8467±0.0875 | 0.7853±0.0735 | 0.8112±0.0621 | 0.5695±0.1574 | 0.5781±0.1534 |
|  | ASDC | 0.7647±0.1289 | 0.7794±0.0763 | 0.8700±0.1343 | 0.7562±0.1229 | 0.8010±0.1045 | 0.5187±0.2644 | 0.5417±0.2604 |
|  | PseDNC | 0.7471±0.0746 | 0.8072±0.0791 | 0.7822±0.0838 | 0.7688±0.0884 | 0.7701±0.0612 | 0.4882±0.1527 | 0.4953±0.1516 |
|  | PseKNC | 0.6824±0.0991 | 0.7291±0.1149 | 0.6856±0.1625 | 0.7198±0.0997 | 0.6917±0.1190 | 0.3654±0.1944 | 0.3732±0.1929 |
|  | ENAC | 0.5765±0.1460 | 0.5661±0.1639 | 0.5767±0.1835 | 0.6211±0.1619 | 0.5891±0.1566 | 0.1504±0.2922 | 0.1554±0.2973 |
| M_831 | NCP | 0.9579±0.0231 | 0.9935±0.0066 | 0.9147±0.0466 | 1.0000±0.0000 | 0.9548±0.0258 | 0.9157±0.0463 | 0.9197±0.0425 |
|  | DBE | 0.9519±0.0210 | 0.9932±0.0069 | 0.9025±0.0423 | 1.0000±0.0000 | 0.9482±0.0241 | 0.9036±0.0420 | 0.9084±0.0378 |
|  | ENAC | 0.9579±0.0231 | 0.9935±0.0066 | 0.9147±0.0466 | 1.0000±0.0000 | 0.9548±0.0258 | 0.9157±0.0463 | 0.9197±0.0425 |
|  | binary | 0.9579±0.0231 | 0.9935±0.0066 | 0.9147±0.0466 | 1.0000±0.0000 | 0.9548±0.0258 | 0.9157±0.0463 | 0.9197±0.0425 |
|  | DPCP2 | 0.9768±0.0163 | 0.9963±0.0061 | 0.9530±0.0329 | 1.0000±0.0000 | 0.9757±0.0173 | 0.9535±0.0326 | 0.9550±0.0312 |
|  | Kmer | 0.7129±0.0469 | 0.7735±0.0435 | 0.7456±0.0585 | 0.6949±0.0434 | 0.7189±0.0480 | 0.4261±0.0938 | 0.4278±0.0941 |
|  | KNN | 0.9398±0.0233 | 0.9911±0.0072 | 0.8798±0.0461 | 0.9980±0.0060 | 0.9345±0.0271 | 0.8794±0.0467 | 0.8862±0.0420 |
|  | Z144 | 0.9038±0.0283 | 0.9557±0.0213 | 0.8851±0.0348 | 0.9172±0.0292 | 0.9007±0.0293 | 0.8074±0.0567 | 0.8082±0.0565 |
| H_3000 | NCP | 0.8997±0.0087 | 0.9669±0.0040 | 0.8057±0.0179 | 0.9966±0.0051 | 0.8909±0.0105 | 0.8001±0.0172 | 0.8157±0.0146 |
|  | ENAC | 0.8940±0.0082 | 0.9642±0.0052 | 0.8099±0.0165 | 0.9782±0.0114 | 0.8860±0.0095 | 0.7886±0.0163 | 0.8011±0.0152 |
|  | binary | 0.8997±0.0087 | 0.9669±0.0040 | 0.8057±0.0179 | 0.9966±0.0051 | 0.8909±0.0105 | 0.8001±0.0172 | 0.8157±0.0146 |
|  | DBE | 0.8964±0.0105 | 0.9663±0.0058 | 0.8085±0.0211 | 0.9854±0.0108 | 0.8880±0.0126 | 0.7934±0.0209 | 0.8071±0.0187 |
|  | PS2 | 0.8985±0.0102 | 0.9641±0.0061 | 0.8375±0.0182 | 0.9579±0.0182 | 0.8935±0.0129 | 0.7975±0.0114 | 0.8042±0.0198 |
|  | KNN | 0.8921±0.0078 | 0.9591±0.0054 | 0.8488±0.0106 | 0.9333±0.0140 | 0.8890±0.0079 | 0.7845±0.0156 | 0.7880±0.0161 |

## Table S8. The l0-fold CV results of different ensemble methods.

| **Dataset** | **Ensemble method** | **ACC** | **MCC** | **Recall** | **Precision** | **F1** | **AUC** |
| --- | --- | --- | --- | --- | --- | --- | --- |
| H_3000 | Ensemb1 | 0.8990±0.0122 | 0.8002±0.0243 | 0.8656±0.0200 | 0.9279±0.0190 | 0.8954±0.0127 | 0.9680±0.0072 |
|  | Ensemb2 | 0.9074±0.0139 | 0.8199±0.0277 | 0.8518±0.0181 | 0.9584±0.0173 | 0.9018±0.0148 | 0.9705±0.0077 |
|  | **Ensemb3** | 0.9081±0.0163 | 0.8252±0.0305 | 0.8346±0.0267 | 0.9781±0.0108 | 0.9005±0.0186 | 0.9713±0.0062 |
|  | Ensemb4 | 0.9064±0.0141 | 0.8218±0.0264 | 0.8332±0.0248 | 0.9760±0.0112 | 0.8988±0.0161 | 0.9703±0.0056 |
|  | Ensemb5 | 0.9062±0.0132 | 0.8175±0.0263 | 0.8513±0.0190 | 0.9564±0.0177 | 0.9006±0.0141 | 0.9705±0.0062 |
| M_831 | **Ensemb1** | 0.9914±0.0086 | 0.9831±0.0168 | 0.9831±0.0168 | 1.0000±0.0000 | 0.9914±0.0086 | 0.9976±0.0033 |
|  | Ensemb2 | 0.9914±0.0086 | 0.9831±0.0168 | 0.9848±0.0175 | 0.9983±0.0050 | 0.9914±0.0086 | 0.9961±0.0064 |
|  | Ensemb3 | 0.9905±0.0081 | 0.9814±0.0158 | 0.9831±0.0168 | 0.9983±0.0050 | 0.9906±0.0081 | 0.9961±0.0062 |
|  | Ensemb4 | 0.9794±0.0154 | 0.9593±0.0305 | 0.9680±0.0231 | 0.9915±0.0138 | 0.9795±0.0154 | 0.9928±0.0072 |
|  | Ensemb5 | 0.9897±0.0075 | 0.9797±0.0146 | 0.9831±0.0168 | 0.9967±0.0067 | 0.9897±0.0075 | 0.9953±0.0074 |
| D_125 | Ensemb1 | 0.7882±0.0840 | 0.5859±0.1739 | 0.9000±0.0778 | 0.7539±0.0730 | 0.8190±0.0675 | 0.8167±0.0802 |
|  | Ensemb2 | 0.7765±0.0686 | 0.5597±0.1421 | 0.8778±0.0778 | 0.7477±0.1589 | 0.8060±0.0582 | 0.8208±0.0747 |
|  | Ensemb3 | 0.7706±0.0718 | 0.5511±0.1511 | 0.8778±0.0923 | 0.7397±0.0581 | 0.8012±0.0637 | 0.8167±0.0809 |
|  | Ensemb4 | 0.7824±0.0689 | 0.5751±0.1455 | 0.8889±0.0861 | 0.7497±0.0590 | 0.8117±0.0608 | 0.8222±0.0889 |
|  | **Ensemb5** | 0.7882±0.0706 | 0.5862±0.1464 | 0.8889±0.0861 | 0.7570±0.0603 | 0.8159±0.0613 | 0.8236±0.0776 |

Note:

H_3000*:* NCP

Ensemble1: Catboost, ET; Ensemble2: Catboost, ET (Catboost as a meta-model); Ensemble3: ET, RF (ET as a meta-model); Ensemble4: ET, RF, and LR (ET as a meta-model); Ensemble5: ET, RF, and Catboost (Catboost as a meta-model)

M_831: NCP

Ensemble1: Catboost, ET (ET as a meta-model); Ensemble2: Catboost, ET (Catboost as a meta-model); Ensemble3: ET, Catboost, LR (Catboost as a meta-model); Ensemble4: ET, Catboost, LR, LDA; Ensemble5: ET, Catboost, LR, LDA (Catboost as a meta-model)

D_125*:* CKSNAP

Ensemble1: Catboost, LR; Ensemble2: ET, LR; Ensemble3: Catboost, ET, LR (Catboost as a meta-model); Ensemble4: Catboost, ET, LR, Ridge (Catboost as a meta-model); Ensemble5: Catboost, ET, LR, Ridge

## Table S9. The 10-fold CV results of different encoding combinations.

| **Dataset** | **Combination method** | **ACC** | **MCC** | **Recall** | **Precision** | **F1** | **AUC** |
| --- | --- | --- | --- | --- | --- | --- | --- |
| H_3000 | **Combin1** | 0.9124±0.0121 | 0.8331±0.0221 | 0.8435±0.0258 | 0.9788±0.0135 | 0.9058±0.0141 | 0.9739±0.0051 |
|  | Combin2 | 0.9083±0.0143 | 0.8276±0.0264 | 0.8373±0.0282 | 0.9786±0.0142 | 0.9021±0.0165 | 0.9732±0.0069 |
|  | Combin3 | 0.9119±0.0167 | 0.8322±0.0302 | 0.8430±0.0333 | 0.9781±0.0100 | 0.9051±0.0193 | 0.9740±0.0059 |
| M_831 | **Combin1** | 0.9931±0.0101 | 0.9865±0.0195 | 0.9864±0.0198 | 1.0000±0.0000 | 0.9931±0.0102 | 0.9975±0.0050 |
|  | Combin2 | 0.9923±0.0105 | 0.9848±0.0204 | 0.9864±0.0198 | 0.9983±0.0051 | 0.9922±0.0107 | 0.9973±0.0055 |
| D_125 | Combin1 | 0.6941±0.0904 | 0.3903±0.1806 | 0.7389±0.1407 | 0.7010±0.0859 | 0.7122±0.0943 | 0.8028±0.1223 |
|  | Combin2 | 0.6647±0.0986 | 0.3302±0.1973 | 0.7056±0.1492 | 0.6672±0.0935 | 0.6821±0.1116 | 0.7639±0.0921 |
|  | **Combin3** | 0.7000±0.0850 | 0.4015±0.1705 | 0.7611±0.1362 | 0.6949±0.0756 | 0.7219±0.0935 | 0.7778±0.1192 |

Note:

**H_3000**:

Combination1: NCP, ENAC, binary, PS2; Combiantion2: NCP, binary; Combination3: NCP, binary, ENAC

**M_831**:

Combination1: NCP, ENAC, binary, DBE, and DPCP2 (default parameters); Combination2: NCP, ENAC, binary, DBE and DPCP2

**D_125**:

Combination1: CKSNAP, Kmer, ASDC, DPCP, PseKNC; Combination2: CKSNAP, ENAC, Kmer, DPCP, PseKNC; Combination3: CKSNAP, Kmer, ASDC, DPCP, PseDNC

## Table S10. The comparison of two incremental feature selection methods across three species.

| **Dataset** | **Stacking method** | **Feature number** | **MCC** | **ACC** | **Recall** | **Precision** | **F1** | **AUC** |
| --- | --- | --- | --- | --- | --- | --- | --- | --- |
| H_3000 | Strategy1 | 443 | 0.8163 | 0.9034 | 0.8291 | 0.9754 | 0.8963 | 0.9713 |
|  | **Strategy2** | 370 | 0.8204 | 0.9062 | 0.8379 | 0.9719 | 0.8999 | 0.9697 |
|  | Original | 1357 | 0.8239 | 0.9056 | 0.8203 | 0.9907 | 0.8975 | 0.9716 |
| M_831 | **Strategy1** | 163 | 0.9841 | 0.9920 | 0.9834 | 1.0000 | 0.9916 | 0.9998 |
|  | Strategy2 | 322 | 0.9841 | 0.9920 | 0.9834 | 1.0000 | 0.9916 | 0.9998 |
|  | Original | 767 | 0.9841 | 0.9920 | 0.9834 | 1.0000 | 0.9916 | 0.9998 |
| D_125 | **Strategy1** | 182 | 0.6191 | 0.8108 | 0.8250 | 0.8250 | 0.8250 | 0.8868 |
|  | Strategy2 | 46 | 0.5930 | 0.7973 | 0.8750 | 0.7778 | 0.8235 | 0.8853 |
|  | Original | 198 | 0.5930 | 0.7973 | 0.8000 | 0.8205 | 0.8101 | 0.8735 |

## Table S11. Performance comparison with the existing tools on the independent test dataset.

| **Dataset** | **Tool** | **Number of correctly identified sites** |
| --- | --- | --- |
| D_300 | PAI | 247 |
|  | EPAI-NC | 253 |
|  | **TIGER** | **254** |

## Table S12. Performance evaluation results of ATTIC on *H. sapiens*, *M. musculus* and *D. melanogaster* datasets

| **Species** | **Experiment** | **MCC** | **ACC** | **Recall** | **Precision** | **F1** | **AUC** |
| --- | --- | --- | --- | --- | --- | --- | --- |
| *H. sapiens* | 10-fold CV | 0.8445±0.0123 | 0.9200±0.0063 | 0.8709±0.0131 | 0.9671±0.0108 | 0.9163±0.0069 | 0.9779±0.0064 |
|  | independent test | 0.8236 | 0.9106 | 0.8679 | 0.9459 | 0.9052 | 0.9711 |
| *M. musculus* | 10-fold CV | 0.9813±0.0159 | 0.9905±0.0081 | 0.9897±0.0158 | 0.9916±0.0111 | 0.9905±0.0082 | 0.9947±0.008 |
|  | independent test | 0.9720 | 0.9860 | 0.9797 | 0.9918 | 0.9857 | 0.9894 |
| *D. melanogaster* | 10-fold CV | 0.5642±0.1516 | 0.7706±0.0765 | 0.8972±0.1083 | 0.7362±0.0809 | 0.8018±0.0608 | 0.8153±0.074 |
|  | independent test | 0.5752 | 0.7838 | 0.8649 | 0.7442 | 0.800 | 0.8692 |

##
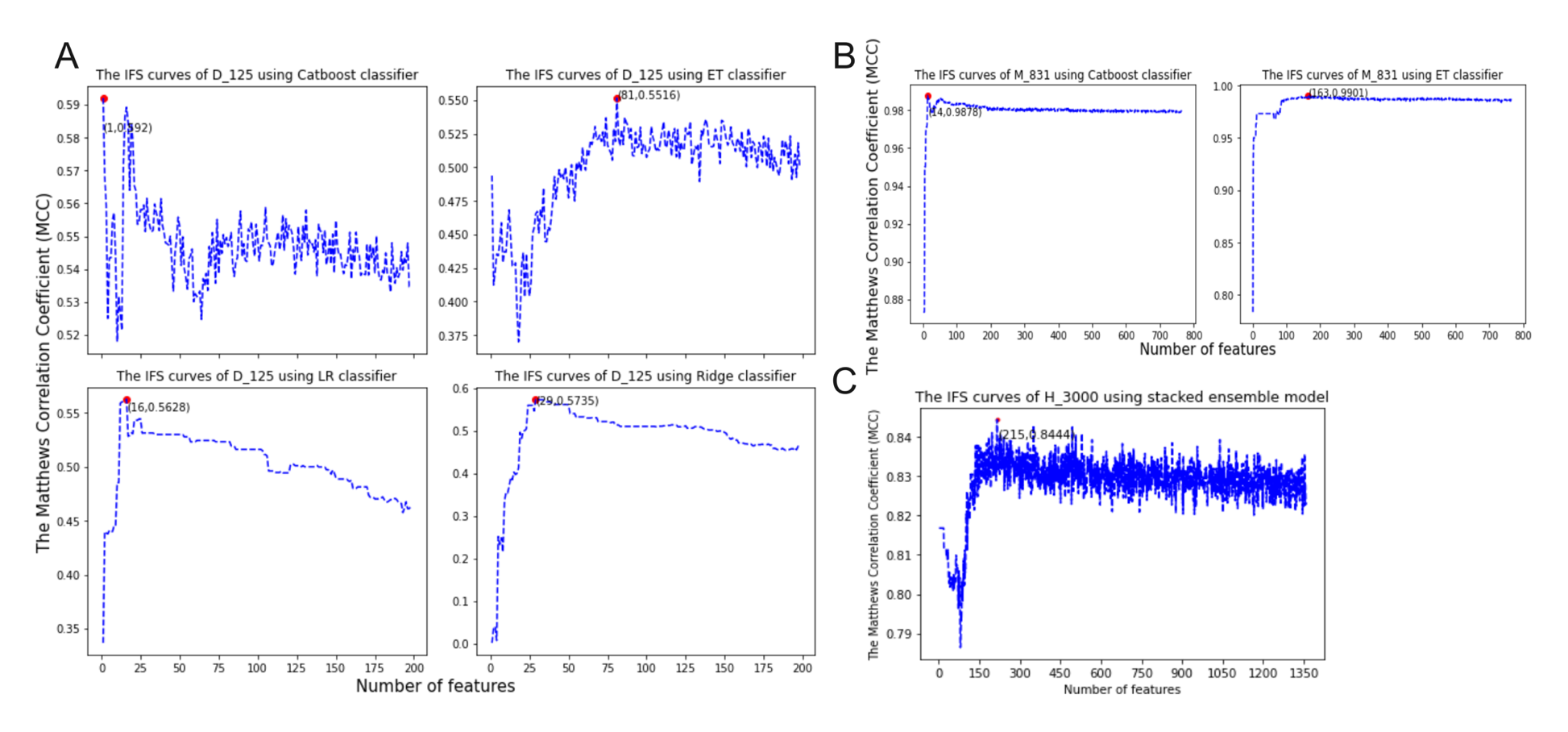
Fig S1. The IFS curves of three benchmark datasets using two incremental feature selection methods. (A) *D. melanogaster*; (B) *M.* *musculus*; (C) *H. sapiens.*

**References:**

1. Chen X, Qiu J-D, Shi S-P, et al. Incorporating key position and amino acid residue features to identify general and species-specific Ubiquitin conjugation sites. Bioinformatics 2013; 29:1614–1622

2. Liu B, Gao X, Zhang H. BioSeq-Analysis2.0: an updated platform for analyzing DNA, RNA and protein sequences at sequence level and residue level based on machine learning approaches. Nucleic Acids Research 2019; 47:e127–e127

3. Chen Z, Liu X, Zhao P, et al. *iFeatureOmega:* an integrative platform for engineering, visualization and analysis of features from molecular sequences, structural and ligand data sets. Nucleic Acids Research 2022; 50:W434–W447

4. Gao F, Zhang C-T. Comparison of various algorithms for recognizing short coding sequences of human genes. Bioinformatics 2004; 20:673–681

5. School of Computational Sciences S.R.T.M.University ,Nanded, MS, 431606, India, Al-Shaibani SAS. A Framework for Implementing Prediction Algorithm over Cloud Data as a Procedure for Cloud Data Mining. JIEEE 2021; 2:1–8

6. Chen W, Xing P, Zou Q. Detecting N6-methyladenosine sites from RNA transcriptomes using ensemble Support Vector Machines. Sci Rep 2017; 7:40242

7. Hasan MdM, Manavalan B, Khatun MstS, et al. i4mC-ROSE, a bioinformatics tool for the identification of DNA N4-methylcytosine sites in the Rosaceae genome. International Journal of Biological Macromolecules 2020; 157:752–758

8. Geurts P, Ernst D, Wehenkel L. Extremely randomized trees. Mach Learn 2006; 63:3–42

9. Park H-A. An Introduction to Logistic Regression: From Basic Concepts to Interpretation with Particular Attention to Nursing Domain. J Korean Acad Nurs 2013; 43:154

10. . Advances in neural information processing systems 27: 28th Annual Conference on Neural Information Processing Systems 2014 [(NIPS)] ; December 8 - 13, 2014, Montreal, Canada ; [proceedings of the 2014 conference]. 2015;

11. Yu C-H, Gao F, Wen Q. An improved quantum algorithm for ridge regression. IEEE Trans. Knowl. Data Eng. 2019; 1–1

12. Xanthopoulos P, Pardalos PM, Trafalis TB. Linear Discriminant Analysis. Robust Data Mining 2013; 27–33
